# Chemistry and properties of fluorescent pyrazole derivatives: an approach to bioimaging applications†

**DOI:** 10.1039/d4ra07485h

**Published:** 2024-12-11

**Authors:** Santiago Melo-Hernández, María-Camila Ríos, Jaime Portilla

**Affiliations:** a Bioorganic Compounds Research Group, Department of Chemistry, Universidad de Los Andes Carrera 1 No. 18A-10 Bogotá 111711 Colombia jportill@uniandes.edu.co

## Abstract

Fluorescent bioimaging is a crucial technique for *in vivo* studies in real cell samples, providing vital information about the metabolism of ions or molecules of biological and pharmaceutical significance. This technique typically uses probes based on organic small-molecule fluorophores, with N-heteroaromatic scaffolds playing an essential role due to their exceptional electronic properties and biocompatibility. Among these, pyrazole derivatives stand out as particularly promising due to their high synthetic versatility and structural diversity. This review highlights prominent examples from the period 2020–2024, focusing on the chemistry, properties, and bioimaging applications of fluorescent pyrazole derivatives. By highlighting the latest advancements in this field, this manuscript aims to inspire and motivate researchers, emphasizing the potential impact of this work on the future of bioimaging.

## Abstract 2

This review considers the latest advancements (2020 to 2024, five articles per year) in the development of fluorescent probes, focusing on the synthesis, chemistry, and properties of pyrazole derivatives for bioimaging applications.

## Graphical abstract

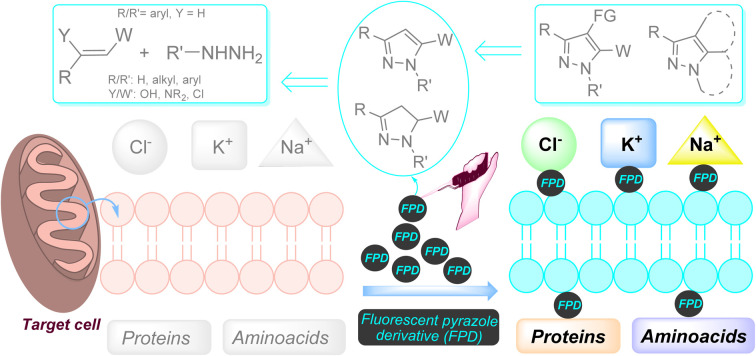

## 1 Introduction

**Fig. 1:**
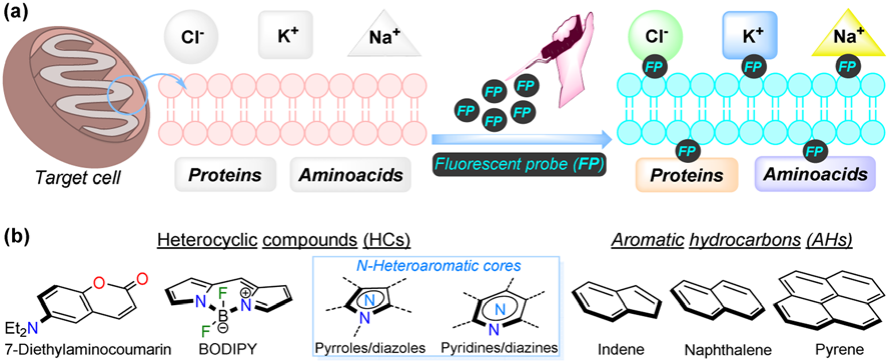
(a) Pictorial depiction of bioimaging acquisition by fluorescent probes. (b) Structural cores of several fluorophores used for bioimaging.

In recent years, non-invasive optical techniques, such as fluorescence-based bioimaging, have emerged as rapid and efficient tools for real-time monitoring of biological processes in live tissues and cells.^1–3^ These methods are crucial for both basic biological research and clinical applications, including therapeutic and diagnostic purposes, as they enable the testing of metabolic processes and the monitoring of the changes in biochemical indicators and biomarkers. The obtained results can be achieved using affordable and user-friendly devices such as fluorescence and confocal microscopes.^4–6^ Applications of these methods include general cell staining,^7–9^ labelling of subcellular structures,^10–12^ and the detection of ions or small molecules, such as Cu^2+^, reactive oxygen species, and reactive nitrogen species (ROS or RNS).^13–17^ These techniques also facilitate the assessment of intracellular conditions, including pH^18–20^ and hypoxia,^21–23^ which are important for sensing cancer,^24–26^ bacterial infections,^27–29^ ischemic injury,^30–32^ and even for theragnostics (a combination of therapy and diagnostics).^33–35^ When a bioimage is required for detection, the sensing can be achieved by monitoring changes in fluorescence, either a turn-on or turn-off response, resulting from the interaction of the probe with the target analyte or cells^1,2,4,5^ (Fig. 1a).

Considering the broad scope of applicability described above, there has been significant interest from both academia and industry in developing suitable systems, platforms, and methodologies for bioimaging applications.^1–35^ However, it remains challenging to identify novel, highly sensitive, and selective probes with good physiological compatibility.^36–38^ Many fluorescent scaffolds have been explored to develop new molecular probes tailored for bioimaging in this field. Various reported fluorophores include those containing heterocyclic rings, such as coumarin,^39,40^ boron complexes (*e.g.*, BODIPY),^41,42^ and N-heteroaromatic cores (*e.g.*, pyrroles, diazoles, pyridines, diazines, *etc.*).^17,43,44^ Additionally, probes based on aromatic hydrocarbons (*e.g.*, indane, naphthalene, pyrene, tetraphenylethene, among others) have also been explored, despite their relatively lower biocompatibility^34,45–47^ (Fig. 1b).

**Fig. 2:**
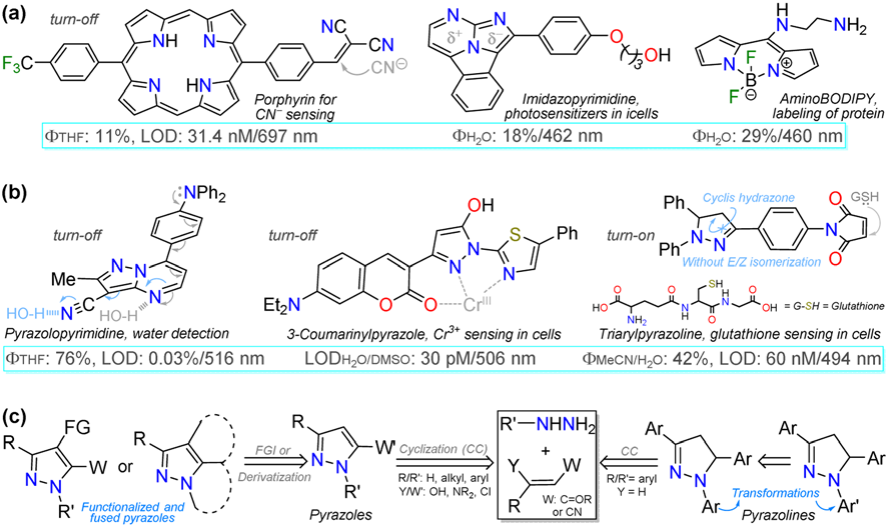
(a) Structure of some fluorescent N-heterocyclic dyes, (b) applications in detection chemistry, and (c) general syntheses of pyrazole derivatives.

Fluorophores based on N-heteroaromatic cores have been extensively explored for various biological and photophysical applications, as the heteroatom imparts important electronic properties to the probes. These compounds are usually stable^17,43,44^ and exhibit excellent synthetic versatility, particularly in terms of ring construction and subsequent functionalization reactions.^43,44,48,49^ Thus, exploration of fluorescent N-heterocyclic compounds remains an ongoing challenge, with continuous efforts aimed at improving their scope and applicability. Many organic compounds have been widely studied in the field of organic materials in recent years,^43,50,51^ leading to the successful application of porphyrins,^52,53^ poly-pyrroles,^54^ boron dipyrrolomethane (BODIPY),^55,56^ and certain 5 : 6 aza-fused diazoles with key dipolar motifs^57–59^ (Fig. 2a). Specifically, diverse pyrazole derivatives have shown fluorescent properties with high quantum yields and good photostability, thermostability, solvatofluorochromism, *etc.*;^38,60–62^ however, what stands out most in pyrazole derivatives is their high synthetic versatility, from the construction of the simple and fused ring to the functionalization reactions of the same^43,63–65^ (Fig. 2b).

Considering bioimaging applications, pyrazole derivatives have been reported to have good membrane permeability and biocompatibility, making them suitable as bioactive and biosensing compounds, and, due to their N-donor character,^60–65^ also ideal for cation detection *in vivo*.^17,62,66^ Many fluorescent pyrazoles for bioimaging purposes have been reported, with the pyrazoline ring being more frequent than the aromatic core; still, their fluorescent property is usually based on their substituents or fused rings (Fig. 2b). Therefore, and as a follow-up to the last review by our group in this regard,^17^ this review covers the chronological reports made in the previous five years (2020–2024) on pyrazole derivatives for use in bioimaging. The discussion focuses on analysing five works per year, covering the compounds' syntheses and their bioimaging properties.

To better understand the purpose of this review, Table 1 presents a summary with the most pertinent results for some of the probes analysed. Specifically, five examples matching each of the years explored are shown, in which the syntheses and applications date are detailed. Table 1 and Fig. 2c show how pyrazoline, pyrazole, and fused pyrazole rings are built by cyclization reactions of 1,3-biselectrophilic reagents with hydrazine derivatives; likewise, the appropriate functionalization or derivatization of pyrazoles for different applications is also shown. The primary aim of this work is to share the knowledge gained on the chemistry of pyrazole derivatives and their photophysical pertinency.^17,43,44,62–65^ Thus, we hope that this review manuscript will be a helpful contribution to further researchers aiming for novel fluorophores synthesis for bioimaging applications.

**Table 1:** Overview of some pyrazole derivatives for bioimaging application of 2020 to 2024^a^

Section, Probe	General probe synthesis	Application data
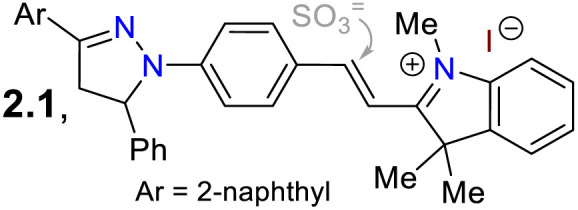	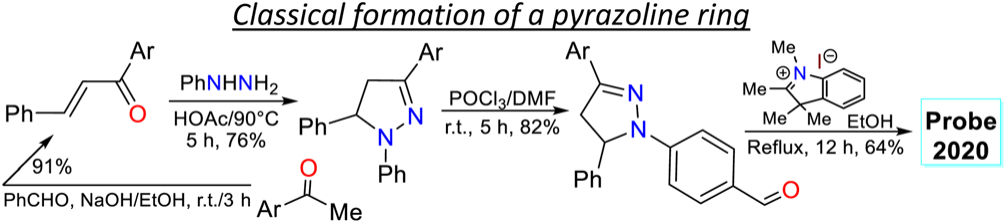	LOD/turn-on = 80 nM at 480 nm in 5% PBS/DMF, *Ф* = 13% at 640 nm.^67^ Mitochondrial sensing
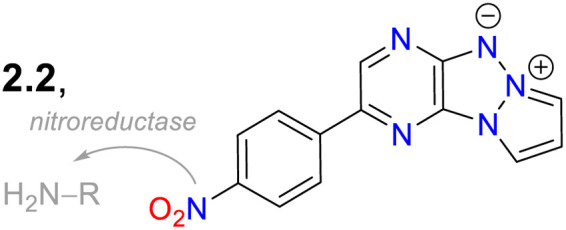	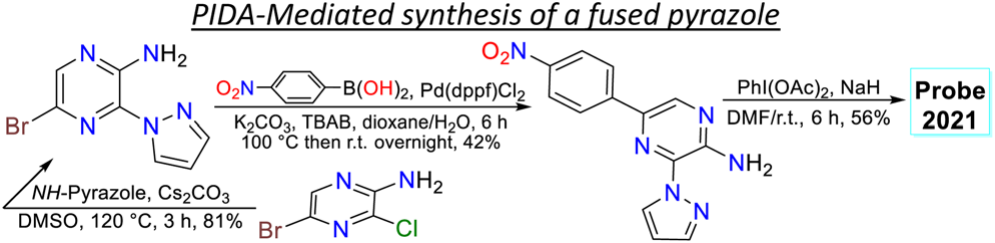	LOD/turn-on = 18.6 ng L^−1^, *Ф* = 76%/R-NH_2_ at 546 nm in DMSO, *τ* = 6.65 ns.^68^ Hypoxia sensing
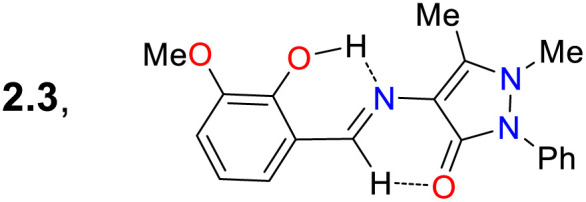	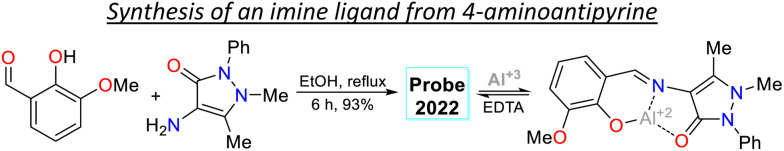	LOD/turn-on = 720 nM at 518 nm in EtOH.^69^ Al^+3^ sensing in human colon living cells (HP-29)
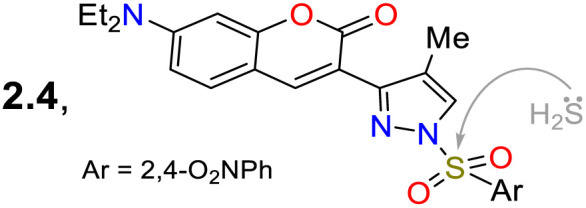	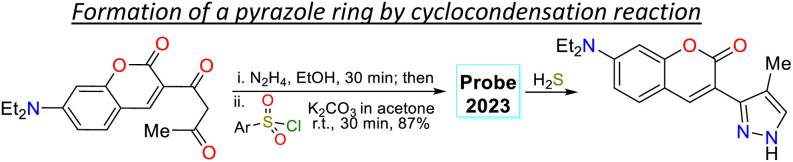	LOD/turn-on = 18.7 nM, *Ф* = 27% at 500 nm in MeCN.^70^ H_2_S sensing in MCF-7 cells
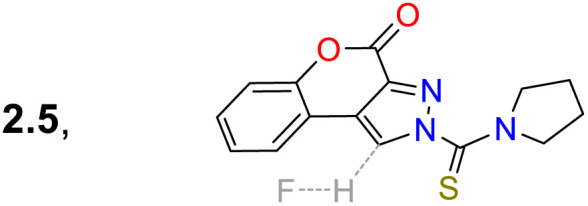	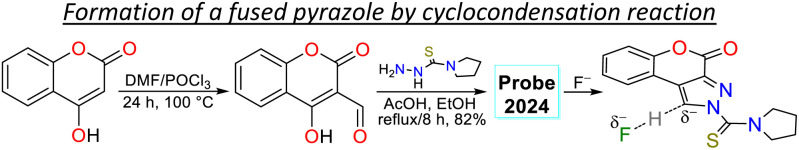	LOD = 4.62 nM/turn-on at 492 nm in MeCN.^71^ F^−^ sensing in T24 cells

a Complete reaction conditions and yields are shown, which is not always common in article schematics.

## 2 Articles analysed

### 2.1. Probes reported in 2020

**Scheme 1:**
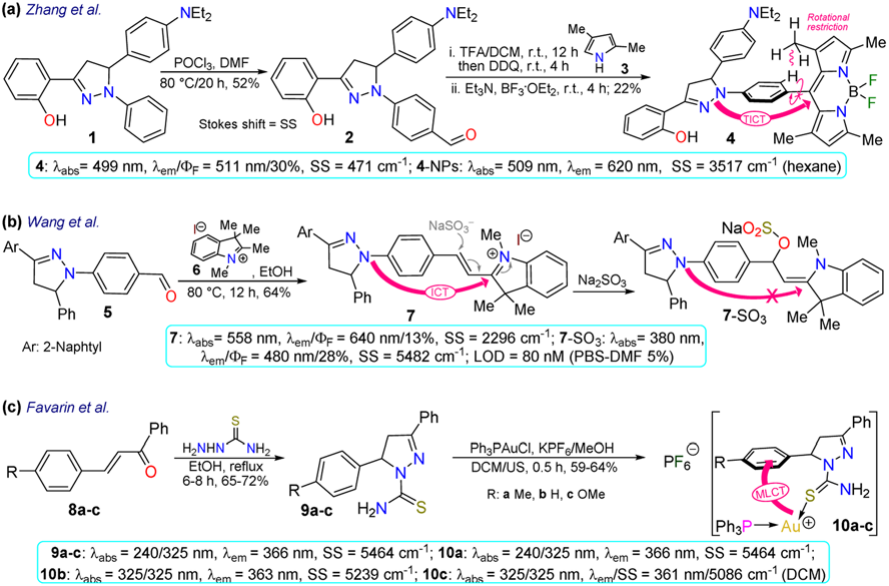
Synthesis and optical data of pyrazolines (a) 4, (b) 7, and (c) 10a–c.

First, Zhang *et al.*^67^ reported the synthesis and long-term bioimaging of the pyrazoline-BODIPY hybrid probe 4 and nanoparticles (NPs) 4-NPs for ultrafast cell staining. Probe 4 was obtained in a 22% yield by the Vilsmeier–Haack reaction of *N*-phenylpyrazoline 1, yielding the *N*-(4-formylphenyl)pyrazoline 2. Then, a one-pot reaction of 2 with 2,4-dimethylpyrrole (3) and BF_3_ offered 4. The probe exhibited an absorption band (*λ*_abs_) at 499 nm and an emission band (*λ*_em_) at 511 nm with a fluorescence quantum yield (*Φ*_F_) of 30%, attributed to an intramolecular charge transfer (ICT) from the pyrazoline ring to the BODIPY ring. The solvatofluorochromism of 4 showed a blue-shift with a decreased fluorescence intensity in polar solvents; however, an opposite result was observed in non-polar solvents. The rotational restriction around the BODIPY ring indicated a twisted ICT (TICT)-locally excited-state effect, which could explains their findings. This probe showed a high *Φ*_F_ in aqueous medium due to an aggregation-induced emission (AIE) process, and it was applied to obtain NPs that were tested on HeLa and A549 cells, which revealed that the fluorescence intensity had a positive dependency on the time of incubation and concentration of 4-NPs. The *in vivo* fluorescence of the probe injected in the tumours of a mouse model was also explored, staining the tumour for up to 12 days, indicating this was probe suitable for long-term, non-invasive tumour progression monitoring (Scheme 1a).

Subsequently, Wang *et al.*^68^ employed probe 7 for *in vivo* sulfite detection in the mitochondria. This probe was synthesised in a 64% yield *via* the condensation of *N*-(4-formylphenyl)pyrazoline with the indolinium salt 6. The *λ*_abs_ at 558 nm and *λ*_em_ at 640 nm with *Φ*_F_ of 13% for 6 showed visible changes in the presence of sulfite by interruption of the ICT process due to a break in the π-conjugation upon the anion addition to the salt, acting as a Michael acceptor. The observed changes showed up as a blueshift to 380 nm and 480 nm with a *Φ*_F_ of 28%, with a reported limit of detection (LOD) of 80 nM for sulfite. This probe was designed to be located in the mitochondria due to the interaction of the negative charge of the mitochondrial membrane and the positive charge of the indolinium salt, which served as the guiding group. Green emission was observed in the presence of sulfite, indicating that it could efficiently sense exogenous and endogenous sulfite (Scheme 1b).

Favarin *et al.*^69^ obtained three gold-based pyrazoline dual probes 10a–c in 59–64% yields by reacting chloro(triphenylphosphine)gold(i) (Ph_3_PAuCl), potassium hexafluorophosphate (KPF_6_), and the pyrazoline-based ligands 9a–c. These ligands exhibited absorption bands around 240 and 325 nm and emission bands around 366 nm, and although they were fluorescent dyes, gold complexes enhanced the emission *via* charge-transfer (CT) processes governed by metal-to-ligand CT (MLCT). The MLCT favoured the intraligand CT (ILCT) and ligand-to-ligand CT (LLCT), as shown by DFT calculations in the ground state, leading the authors to assume that the emission of complexes 10a–c was due to the mentioned CT processes. However, the photophysical mechanism in the excited state still needs to be studied in more depth as this is less simple. The CT processes led to two *λ*_em_, a blue one when excited at about 305 nm and a green one when excited at 405 nm. Cellular imaging was performed on two cancer cell lines (MDA-MD231 and MCF-7) and one non-cancerous healthy cell line (HUVEC). For the studied cell lines, 10a–c showed good internalisation and staining of the cytoplasm and they seemed to be suitable dyes for general staining or even for co-staining assays, as their dual emission may allow using further dyes that are compatible with at least one of the fluorescence modes of 10a–c, thereby increasing their span of utility. However, it must be noted that 10a–c were found to be highly cytotoxic for cancer and non-cancer cell lines, which would impair their application for *in vivo* imaging, as their use would hinder studying cellular processes and lead to the death of the cells (Scheme 1c).

**Scheme 2:**
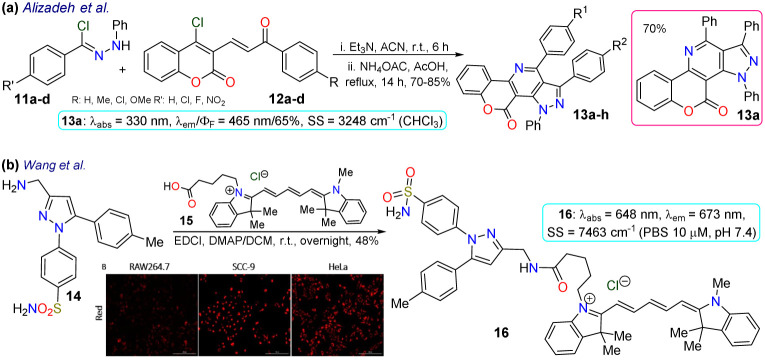
Synthesis and photophysical data of (a) the fused pyrazoles 13a–h and (b) the π-extended pyrazole 16. Bioimage of 16 in RAW264.7, SCC-9, and HeLa cell lines by ref. 71 with minor modifications licensed under Creative Commons CC BY 4.0.

Alizadeh *et al.*^70^ used the pyrazolopyridine–coumarin hybrid probes 13a–h for general cell staining, synthesised in 70–85% yields *via* a 1,3-dipolar cycloaddition reaction of hydrazonoyl chloride 11 and the enone 12. The reaction proceeded in the presence of ammonium acetate by nucleophilic substitution of the chloride on the coumarin moiety with the nitrogen source, forming an enamine that then reacted with the carbonyl to generate products after aromatization. These probes exhibited coplanar, rigid, and aromatic structures, which were responsible for their fluorescence, except for probes bearing a nitro group as this favoured internal conversion over radiative decay; likewise, the emission spectra were barely affected by the substituents. As a representative example, probe 13a exhibited *λ*_abs_ at 330 nm, *λ*_em_ at 465 nm, *Φ*_F_ of 65%, good photostability, and pH sensitivity; as a result, this probe was applied for the cellular imaging of MG-63 cells. The probe showed good internalisation into the cell, apparently even into the nuclei, as there were no black spots on the images after 30 min of incubation (Scheme 2a).

As a final example in 2020, Wang *et al.*^71^ obtained the NIR fluorescent probe celecoxib-based 16 in a 48% yield by the amidation reaction of the 3-aminomethylpyrazole 14 with the carboxylic acid 15 bearing a cyanine moiety. This probe was used in cyclooxygenase sensing 2 (COX-2) in tumoural cells. The photophysical properties of probe 16 were recorded in PBS at pH 7.4 to achieve conditions as close as possible to the cellular environment, displaying *λ*_abs_ at 648 nm and *λ*_em_ at 673 nm. In addition, docking studies showed that 16 was a potent inhibitor of COX-2, whose binding site was the same as free celecoxib, *i.e.* inside the active side pocket. The COX-2 inhibiting activity of 16 was confirmed by *in vitro* tests using purified COX-2m, and its actual capability for imaging in living cells was investigated by imaging assays in a normal cell line (RAW264.7) and two cancer cell lines (HeLa and SCC-9). It's very low toxicity to the three cell lines with differential and scarce fluorescence for normal cells but strong fluorescence for cancer cells was evidenced; thus, 16 could discern normal and cancerous cells. As a result, the optical mechanism for 16 must be associated with its accumulation around COX-2, which is less expressed in normal cells, explaining why the RAW264.7 cells barely shined while the cancer cells strongly fluoresced (Scheme 2b).

### 2.2. Probes reported in 2021

**Scheme 3:**
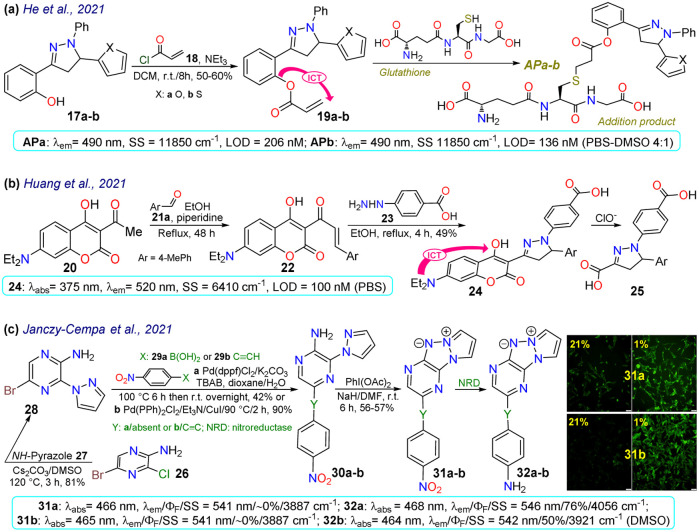
Synthesis and photophysical data of (a) 19a–b, (b) 24, and (c) 31/32a–b. Bioimaging of A2058 cells cultured under normoxic (21% pO_2_) and hypoxic (1% pO_2_) conditions for 24 h with 31a–b (prior 2 h at 8 μM); ref. 73 with minor changes licensed under Creative Commons CC BY 4.0.

He *et al.*^72^ synthesised the (pyrazolin-3-yl) phenyl acrylate 19a–b in a 50–60% yield by the esterification between 2-(pyrazolin-3-yl)phenol 17a–b and acryloyl chloride 18. These probes exhibited very weak emission at 490 nm, but this increased upon the nucleophilic addition of glutathione to the acrylate receptor fragments 19a–b. Utilising DFT studies, the authors reported that the highest occupied molecular orbital (HOMO) was focused on the *N*-phenylpyrazoline moiety and the lowest unoccupied molecular orbital (LUMO) on the acrylate, causing ICT with fluorescence turn-off; thus, the LUMO was disturbed upon the conjugated addition of the respective thiol group, raising the HOMO–LUMO energy gap and thereby preventing quenching. The probes offered low toxicity, and 19a was applied for imaging tests in cells cultured with and without glutathione. Incubation of the cells with 19a led to blue fluorescence inside the cells without any morphological artefacts, indicating that 19a could detect endogenous glutathione, and has potential as a new tool for studying glutathione's biological role in different processes (Scheme 3a).

Huang *et al.*^74^ obtained the coumarin-3-ylpyrazoline 24 for *in vivo* hypochlorite sensing by reacting 3-acetylcoumarin 20 with *p*-tolualdehyde (21a) to produce the enone 22, which then reacted with arylhydrazine 23 to afford 24 in a 49% yield. Probe 24 showed *λ*_abs_ at 375 nm, *λ*_em_ at 520 nm, and a donor–acceptor structure from an electron-donating group (EDG), such as diethylamino, to an electron-withdrawing group (EWG), such as hydrazone, in the pyrazoline fragment, favouring the emission of 24 by an ICT phenomenon. This probe was degraded to the dicarboxylic acid 25 through hypochlorite-mediated oxidation upon adding the analyte (on the coumarin ring), quenching its fluorescence. The fluorophore 24 showed slight cytotoxicity and good internalisation into the cytoplasm for cell imaging. When the cells or zebrafish were cultured without the analyte, the probe showed strong green fluorescence, which was quenched upon addition of the analyte, implying that 24 could detect exogenous ClO^−^. HeLa cells were also incubated with LPS to stimulate the endogenous production of ClO^−^, which was also detected (Scheme 3b).

Janczy-Cempa *et al.*^73^ used the nitrocompounds 31a–b as probes sensitive to nitroreductase, allowing *in vivo* hypoxia evaluation by imaging. The probes were synthesised by reacting the pyrazine 26 with *NH*-pyrazole (27) to form the *N*-hetarylpyrazole 28, which then reacted with the appropriate nitroaryl derivative 29a–b to yield the intermediates 30a–b, which were finally subjected to a PIDA-mediated cyclization reaction (Scheme 3c). Probes 31a–b showed *λ*_abs_ around 465 nm, *λ*_em_ around 541 nm, and very weak fluorescence as the nitro group quenched the process. The reduced fluorophores 32a–b showed good photostability and responses against human nitroreductase (NRD), even in the presence of interferents, and their fluorescence was not affected by molecular oxygen. To evaluate the bioimaging suitability of the probes, they were cultured with A2058 cells under normoxic and hypoxic conditions. Cells grown under normoxic conditions showed a slight fluorescence emission, possibly due to the activity of basal levels of NRD. However, when subjected to hypoxic conditions (less than 1% oxygen), the cells showed increased green emission, indicating a more significant reduction of probes 31a–b.

**Scheme 4:**
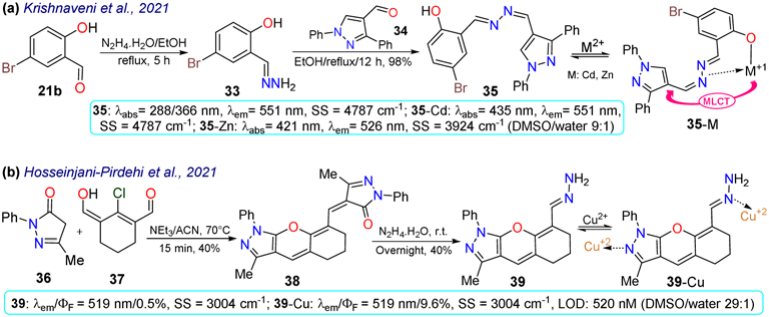
Synthesis and photophysical data of (a) 35/35-M and (b) 39/39-Cu.

Krishnaveni *et al.*^75^ reported bisimine 35 as a probe for the *in vivo* detection of Zn^2+^ that was prepared by the condensation reaction of 5-bromosalicylaldehyde (21b) with hydrazine and then with 4-formylpyrazole 34 to produce the probe in a 98% yield. Probe 35 exhibited two *λ*_abs_ at 288 and 366 nm, a weak *λ*_em_ at 511 nm, a HOMO spread over the entire molecule, and a LUMO focused over the imine-bromophenol moiety. No fluorescence was observed for 35 due to the *s-cis*/*s-trans* isomerisation of the bisimine group (–CH=N–N=CH–). However, when 35 was complexed with Cd^2+^ or Zn^2+^, the HOMO was focused on the metal, while the LUMO remained similar; thus, the fluorescence in 35-M was attributed to a metal-to-ligand charge transfer (MLCT). This probe could detect Zn^2+^ in HeLa cells and zebrafish embryos, exhibiting little toxicity. Zebrafish did not show fluorescence with 35 and without the analyte, while an intense yellow emission was observed under the exposition and internalisation with Zn^2+^ (Scheme 4a).

In the last example of 2021, Hosseinjani-Pirdehi *et al.*^76^ obtained the tetrahydro-chromeno[2,3-*c*]pyrazole 39 by reacting 2 equivalents of pyrazolone 36 with formylated 1,3-biselectrophyle 37 to produce 38, which ultimately suffered hydrazinolysis at 39 in a 40% yield (Scheme 4b). This probe was used for *in vivo* Cu^2+^ sensing and both 39 and 39-Cu showed no displacements of *λ*_abs_ and *λ*_em_, but the fluorescence was enhanced in 39-Cu. For 39, no fluorescence was observed due to the *E*/*Z* isomerisation of the hydrazone moiety, which was restricted once the metal had chelated, causing more rigidity in the molecule. This probe was used for bioimaging and the *in vivo* detection of Cu^2+^. Cells incubated solely with 39 showed no emission; however, the cells showed a greenish-yellow emission after Cu^2+^ was added to the culture. This result indicates that 39 had reasonable internalisation rates and could detect intracellular Cu^2+^. However, it is essential to note that 39 showed significant toxicity towards cells. Given this, 39 might not be the best tool for ion monitoring in living systems, but it could still be used for *in vitro* or *ex vivo* testing.

### 2.3. Probes reported in 2022

**Scheme 5:**
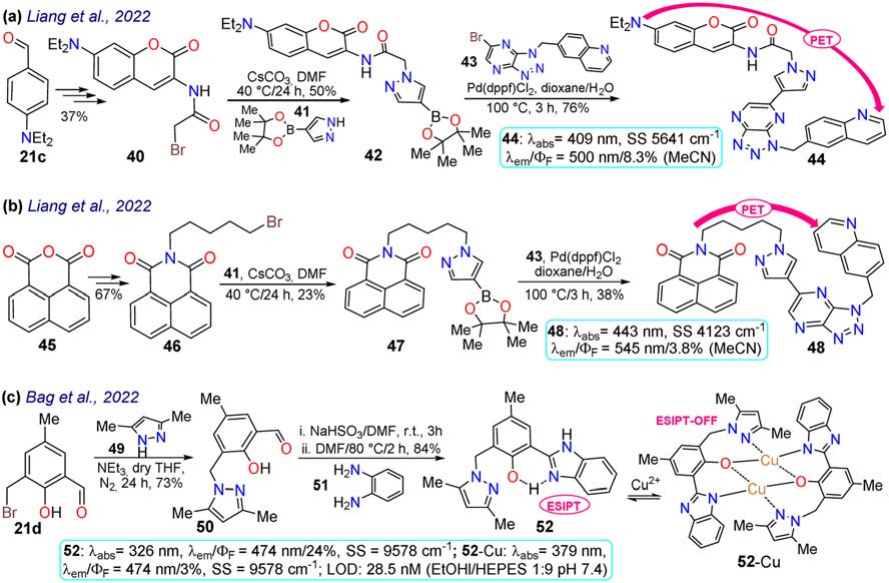
Synthesis and photophysical data of (a) 44, (b) 48, and (c) 52/52-Cu.

Liang *et al.*^37^ obtained the 1,4-disubstituted pyrazoles 44 and 48 for the *in vivo* detection of c-Met, a protein highly expressed in solid tumours that is a strategic target for cancer diagnosis and detection. The synthesis started from the arylaldehyde 21c or anhydride 45, and it continued by incorporating a bromo-alkyl moiety that was then stirred with the pyrazolyl–boronic acid 41 to form the boronic esters 42 and 47, which were subjected to a Suzuki–Miyaura reaction yielding 44 and 48. Both dyes showed a photoinduced electron transfer (PET) process with fluorescence turn-off, in which the excited coumarin moiety transferred an electron to the receptor quinoline moiety; however, this process was inhibited upon binding to c-Met, turning on the emission of 44 and 48 (Schemes 5a and 5b). As for the bioimaging assays, both probes exhibited slight fluorescence in normal cells, which was related to basal levels of c-Met as this is a protein linked to several metabolic processes for almost any cell. In contrast, when the fluorophore was used with a c-Met overexpressing cell line, there was an apparent increase in the fluorescence, indicating that both probes could be used for detecting changes in the levels of c-Met. However, it must be considered that both dyes were highly toxic to at least two cell lines; thus, their application spectrum might not be as broad as expected.

Later, Bag *et al.*^77^ synthesised the pyrazole derivative 52, an excited-state intramolecular proton-transfer (ESIPT) active sensor for sensing Cu^2+^ in cells and plants. Their synthesis started from the 3-bromomethylsalicylaldehyde 21d and the *NH*-pyrazole 49 to form the intermediate 50; which was then reacted with sodium bisulfite and later with *o*-phenylenediamine 51 to yield the benzimidazole derivative 52.^77^ Unlike other sensors for metal cations analysed above, 52 was a turn-off sensor whose emission was quenched by adding Cu^2+^. This dye exhibited a *λ*_abs_ at 326 nm and a *λ*_em_ at 474 nm with a *Φ*_F_ of 24%, whereas 52-Cu showed a redshifted *λ*_abs_ at 379 nm with a *λ*_em_ that was not shifted but was dramatically decreased (*Φ*_F_ = 3%) due to an ESIPT process. In the excited state, the OH group of 52 formed intramolecular hydrogen bonds with pyridine-like nitrogen in pyrazole and benzimidazole rings, giving a strong blue emission; still, the fluorescence was quenched upon the formation of 52-Cu as the heteroatoms were occupied. Next, 52 was used for *in vivo* Cu^2+^ sensing, with MCF-7 cells, chickpeas, and Mung beans tested. For the cells, 52 showed almost no toxicity; while samples treated with 52 without Cu^2+^ showed strong blue emission inside the cells and the sprout of the beans, revealing that 52 could be well absorbed in mammal and plant cells. In contrast, when the samples were treated with 52 and Cu^2+^, the fluorescence was quenched in cells and plants (Scheme 5c).

**Scheme 6:**
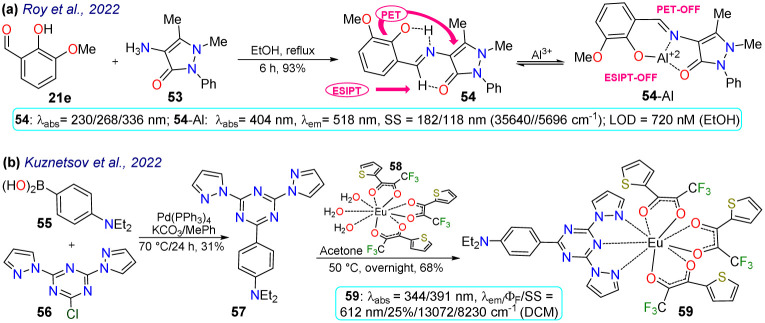
Synthesis and photophysical data of probes (a) 54/54-Al and (b) 59.

Roy *et al.*^78^ obtained the pyrazolone 54 by reacting 3-methoxy-salicylaldehyde (21e) with the aminopyrazolone 53. This probe showed three *λ*_abs_ at 230, 268, and 326 nm but was not fluorescent due to PET and ESIPT processes. However, complex 54-Al showed a redshift to 404 nm. Upon the excitation of 54, its aryloxyimine group transferred one electron to the adjacent enone moiety, and the molecular conformation allowed the intramolecular hydrogen bond formation. Notably, the PET and ESIPT processes were not viable after forming 54-Al as the heteroatom donors were occupied in chelating the Al^3+^ (Scheme 6a). Likewise, 54 was used for intracellular Al^3+^ sensing by bioimaging, with an absence of emission observed for cells treated with Al^3+^, but once the dye was added, a strong green fluorescence was seen. Nevertheless, care must be taken when using this probe as it was confirmed through utilising an acridine orange/ethidium bromide co-staining assay that the 54-Al complex may interact with single-stranded DNA, which could affect the cell cycle and lead to cell death *via* apoptosis.

Finally, Kuznetsov *et al.*^79^ prepared the phosphorescent probe 59 by a Suzuki coupling of the arylboronic acid 55 with the heteroaryl chloride 56, yielding the tridentate ligand 57, which then reacted with the Eu(iii) complex 58, delivering 59. This probe showed two *λ*_abs_ at 344 and 391 nm with a *λ*_em_ at 612 nm, typical for Eu(iii) complexes (Scheme 6b). As NIR radiation is minor phototoxic and more penetrating than visible radiation, these results suggest the probe would not cause cellular photodamage and would offer deep tissue penetration. By testing the temperature effect on the luminescence of 59, it was found that raising the temperature reduced the lifetime of its emission, indicating its high sensitivity to temperature changes. Nanoparticles 59-NPs with a superficial positive charge were also prepared as their target is the mitochondrion, whose membrane is anionic. Still, 59-NPs were found in endosomes, lysosomes, or similar parts, not in the mitochondria. Moreover, phosphorescence lifetime imaging microscopy (PLIM) assays were used with uncharged 59-NPs for sensing cellular temperature. The images showed a general typical temperature in cells, although some colder spots could also be seen. However, these results were good enough to indicate that the probe is suitable and viable for intracellular temperature review. The same behaviour was found for modified 59-NPs, indicating that the cationic surface did not affect its sensing properties.

### 2.4. Probes reported in 2023

**Scheme 7:**
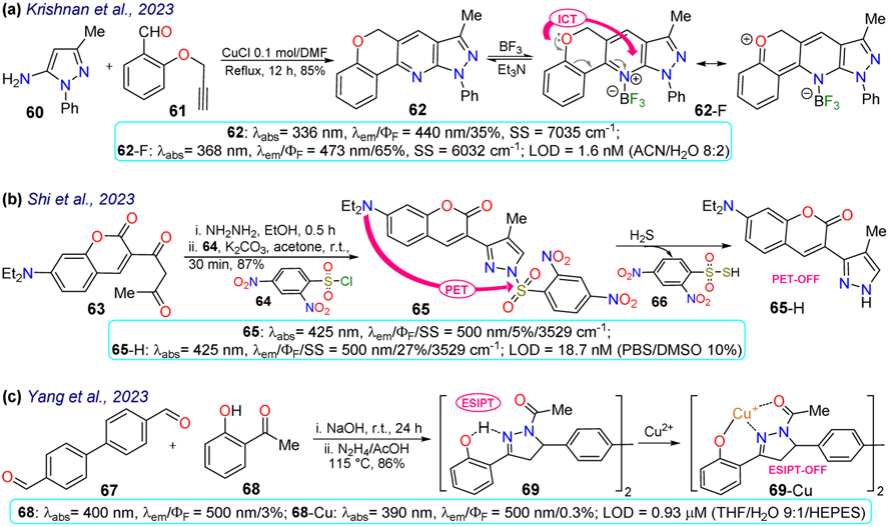
Synthesis and optical data of probes based on (a) pyrazolopyridine 62/62-F, (b) 3-(coumarin-3-yl)pyrazole 65/65-H, and (c) pyrazoline 69/69-Cu.

Krishnan *et al.*^80^ synthesised pyrazolo[4,3-*b*]pyridine 62 in an 85% yield through a Povarouv reaction of 5-aminopyrazole 60 with the arylaldehyde 61 and then used this probe for the detection of BF_3_ in *E. coli* and HeLa cells. Probe 62 displayed an absorption band at 336 nm and an emission band at 440 nm, which were then redshifted in the presence of BF_3_ – at 368 and 473 nm, respectively. Upon gradual BF_3_ addition, the absorption band of probe 62 decreased while the fluorescence band increased, increasing the quantum yield from 35% to 65% due to ICT from the alkoxy group to the boron atom. Probe 62 was tested in the presence of *E. coli* cells, with blue fluorescence observed once the bacterial culture was incubated with BF_3_. Also, 3-(4,5-dimethylthiazol-2-yl)-2,5-diphenyltetrazolium bromide (MTT) assays were performed, offering green fluorescence, which indicates that the intracellular uptake of BF_3_ resulted in the complexation in the intracellular region (Scheme 7a).

In another work, Shi *et al.*^81^ prepared 3-(coumarin-3-yl)pyrazole 65 by two steps in an 87% yield, implying a cyclocondensation reaction of the β-diketone 63 with hydrazine, followed by a sulfonylation reaction with 2,4-dinitrobenzenesulfonyl chloride (64). This probe exhibited a *λ*_abs_ at 425 nm and a *λ*_em_ at 500 nm without a shift in these wavelengths in the presence of H_2_S. Nevertheless, upon analyte addition, the solution changed its colour, and the *Φ*_F_ was enhanced from 5% to 27% as a PET-OFF process was activated once the sulfonyl group was cleaved in 64 by H_2_S. Competitive assays were performed with varied cations, anions, and some amino acids, and it was found that the reaction with H_2_S was unperturbed by the presence of other analytes and lasted 3 min. Still, with other analytes like cysteine, the reaction took 30 min or longer. Ultimately, the authors investigated the recognition of exogenous and endogenous H_2_S in MCF-7 cells, which were incubated with the analyte and a fluorescence turn-on of the cells was observed with an LOD of 18.7 nM (Scheme 7b).

Yang *et al.*^82^ synthesised the bispyrazoline 69 in an 86% yield through a condensation reaction of 4,4′-diformylbiphenyl (67) with 2-hydroxyacetophenone 68 followed by a cyclisation reaction with hydrazine. Probe 69 exhibited a *λ*_abs_ at 400 nm, shifted to 390 nm in the presence of Cu^2+^, and a *λ*_em_ at 500 nm. In the presence of the analyte, the fluorescence decreased, with *Φ*_F_ going from 3% to 0.3% due to an interrupted ESIPT process. Biological assays were performed on HeLa cells with 69 and 69-Cu, evidencing that these cells had blue emission with good permeability in the presence of 69, and then by adding Cu^2+^ to the system, the fluorescence signal weakened, making it suitable for the detection of intracellular copper ions (Scheme 7c).

**Scheme 8:**
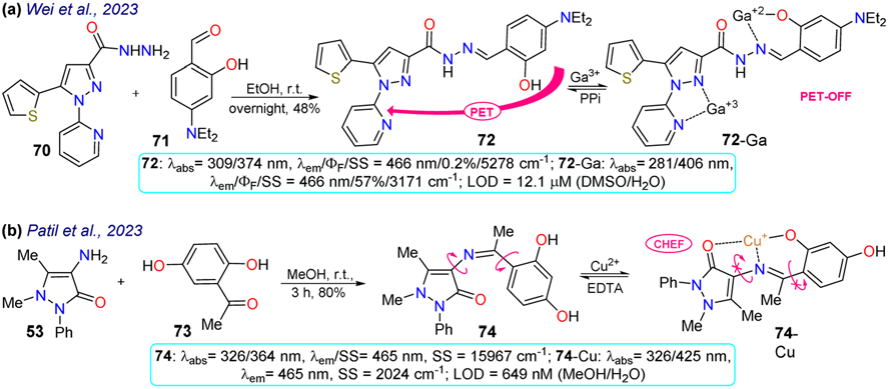
Synthesis and optical data of (a) 72/72-Ga and (b) 74/74-Cu.

Two examples of pyrazoles were reported in 2023 that do not present an approach to bioimaging but focus on the design of fluorophores, as discussed below. First, Wei *et al.*^83^ obtained 72 by condensing 3-pyrazolylhydrazide 70 with salicylaldehyde 71. This probe showed two *λ*_abs_ at 309 and 374 nm, which were shifted to 281 and 406 nm upon the addition of Ga^3+^ ions. Likewise, weak fluorescence was observed (*Φ*_F_ = 0.2%), which was increased (*Φ*_F_ = 57%) in the presence of the analyte, obtaining an LOD of 12.1 mM. The almost no fluorescence of 72 was due to a PET-OFF process in the complex 72-Ga. The authors studied the effect of the diethylamino group in 72, evidencing the necessity of this group for the photophysical properties (Scheme 8a). Second, Patil *et al.*^84^ synthesised the imine 74 in an 80% yield using the reaction of 4-aminoantipyrine (72) with 2,5-dihydroxyacetophenone (73). This dye exhibits two *λ*_abs_ at 326 and 364 nm, but in the presence of Cu^2+^, the band at 364 nm was shifted to 425 nm (Scheme 8b). Upon increasing the concentration of Cu^2+^, a progressive rise and decrease in the peaks at 425 and 364 nm, respectively, was observed, which evidenced the formation of the complex 74-Cu, which favoured a ligand-to-metal charge transfer (LMCT). An emission peak at 465 nm was evidenced for 74 and 74-Cu, with fluorescence enhancement in the presence of Cu^2+^ due to a chelation-enhanced fluorescence process (CHEF).

### 2.5. Probes reported in 2024

**Scheme 9:**
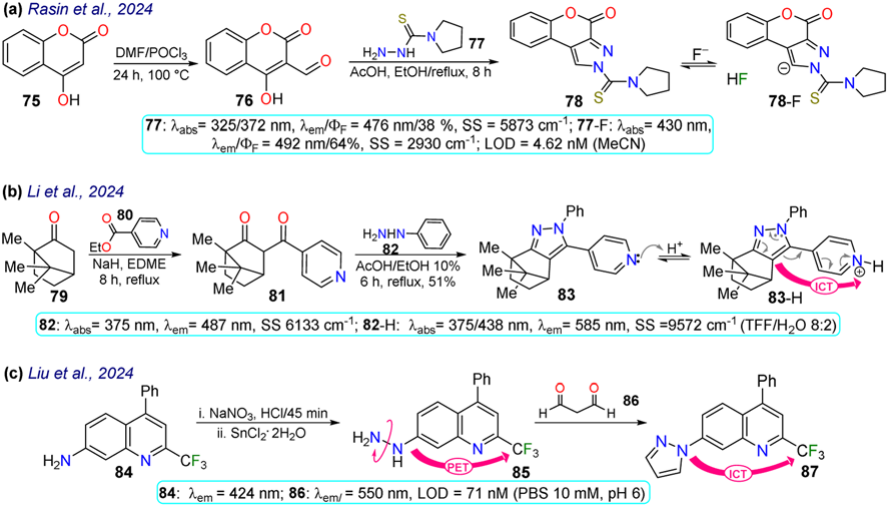
Synthesis and photophysical data of fluorophores (a) 78/78-F, (b) 83/83-H, and (c) 85/87.

Rasin *et al.*^85^ synthesised the fused pyrazole 78 by the cyclisation reaction of the 1,3-biselectrophyle 76 with the thiosemicarbazide 77. Probe 78 exhibited two absorption bands at 325 nm and 372 nm, and upon the addition of fluoride ions, a new band emerged around 430 nm, indicating the formation of 78-F; 78 displayed a *λ*_em_ at 476 nm that was shifted to 492 nm for 78-F with an increase in the *Φ*_F_ from 38% to 64%. Complex 78-F demonstrated a fluorescence enhancement compared to 78, which linked the relaxation from the stimulated state to the PET process. The authors investigated the detection of the anion on T24 living cells, and blue fluorescence was observed once the cells were treated with the sensor and fluoride ions, indicating that 78 could detect the anion on the cell's cytoplasm (Scheme 9a).

Li *et al.*^86^ designed probe 83 to detect aqueous acid, which was obtained using the condensation reaction of the bicyclic ketone 79 with ethyl isonicotinate 80 to form the β-diketone 81, which finally cyclocondensed with phenylhydrazine (82). Probe 83 exhibited an absorption band at 375 nm, which was gradually diminished with the increase in the medium's acidity, and a new peak emerged at 438 nm. Likewise, an emission band at 487 nm was observed that decreased as the acidity increased, while a new peak at 585 nm emerged. These spectral changes were caused by protonation of the pyridine ring generating an ICT effect that led to the redshift of the spectrum. The authors conducted fluorescent bioimage studies, which indicated that 83 exhibited low cytotoxicity, making the probe well suited for monitoring pH alterations of zebrafishes as they show a strong fluorescence in acidic media (Scheme 9b).

Liu *et al.*^87^ obtained the pyrazole 87 by the cyclocondensation reaction of malonaldehyde (86) with the hetarylhydrazine 85, which was obtained *via* diazonium salts from the hetarylamine 84 (Scheme 9c). The 4-trifluoromethylquinoline group of 85 was the Golgi-target, and the hydrazine group was the recognition site. This probe exhibited an emission band at 424 nm but showed no fluorescence, due to the PET process generated by the presence of the amino group. However, once the probe reacted with 86, forming 87, the electron-donating capacity of the amino group was affected, offering both PET-OFF and ICT phenomena with a redshift in the emission spectra and an enhanced fluorescence. The MTT method was used with 87, and low cytotoxicity was found. Then, the ability of 85 to Golgi apparatus was proved by labelling HeLa cells and red staining the Golgi apparatus, with blue emission detected, indicating the excellent targeting of the probe with the desired target, with an LOD of 71 nM.

**Scheme 10:**
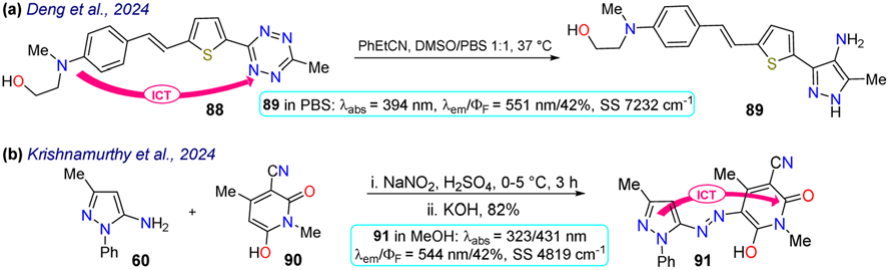
Synthesis and photophysical data of the probes (a) 89 and (b) 91.

Deng and colleagues prepared the probe 89 using the reaction of tetrazine 88 with phenyl-propanenitrile, a type of [4 + 1] cycloaddition that is an alternative to the inverse electron-demanding Diels–Alder reaction that has gained attention in protein labelling, and drug delivery and thanks to its biocompatibility, rapid kinetics, and effective payload release (Scheme 10a).^88^ Probe 89 exhibited absorption and emission bands at 394 and 551 nm, respectively, with a *Φ*_F_ of 42% in PBS buffer, probably giving an ICT-OFF process that initially occurs from the dialkylamino group to the π-deficient tetrazine ring. This probe was studied with live 193T-cell labelling on intracellular targets (nucleus proteins and mitochondria). Vivid mitochondria stains and orthogonal labelling were observed in the membrane, suggesting probe 89 is suitable for labelling bioimages.

Finally, Krishnamurthy *et al.*^89^ synthesised probe 91 in an 82% yield starting from the 5-aminopyrazole 60, which underwent diazotization followed by a coupling reaction with the cyanomethyl-pyridone 90. Probe 91 exhibited two absorption peaks at 323 nm and 431 nm and an emission peak at 544 nm with a *Φ*_F_ of 42% in methanol, which helped stabilise the hydrazo tautomer 91. In solvents such as MeOH, DMSO, and DMF, the hydrazo form was stabilised generating a redshift on the absorption peaks, while in MeCN, DCM, and chloroform the azo form was stabilised generating a blue-shift on the spectra. An ICT process was responsible for the fluorescence of probe 91. This property was used for bioimaging in HeLa cells, with a strong green fluorescence observed, and was studied for the electrochemical detection of dopamine, demonstrating an LOD of 0.81 mM (Scheme 10b).

## 3 Conclusions

In summary, diverse synthetic transformations implying fluorescent pyrazoles have been reported, some by ring construction and others using functionalisation or derivatisation strategies, usually by classical reactions and reaction conditions that are easily reproducible. In this manner, novel functional pyrazoles with relevant applications have been obtained, with some examples finding use in bioimaging applications. These fluorescent dyes have proven to offer exceptional results in general cell staining and the detection of in-cell conditions, like temperature or hypoxia. The compounds herein discussed include further structural motifs, from additional heterocyclic, pyrazolines, or fluorescent groups to complexes with metal ions, for which different synthetic approaches are needed. For the evaluated systems, ICT, PET, and MLCT are the most prevalent photophysical phenomena that govern the fluorescence of the probes, which, upon reacting with the target (like metal ions, small molecules, or enzymes), is either enhanced or ‘turned-off’, leading to observable changes in the fluorescence that allow the sensing of the different targets, which in turn is what leads to pyrazole derivatives having so many applications. Various molecular architectures of pyrazoles-based compounds have been used in bioimaging applications, in which the incorporation of EDGs or highly conjugated substituents is the main constant in the probe design. Also, the probes must be functionalised with appropriate receptors (*e.g.* ion receptors, lipophilic fragments, or hydrogen bond mediators) so that they can perform their work in different environments. Notably, although there are many published articles on fluorescent probes for bioimaging applications based on small molecules, several of these works did not carry out an appropriate photophysical study for the respective development; as a result, a good look at this review could pointedly improve this issue.

## Data availability

This review manuscript presents original work submitted for publication only in RSC Advances. The authors have no conflicts of interest to report with this submission, and they have all seen, revised, and approved this paper, which details the chemistry and properties of fluorescent probes based on pyrazole/pyrazoline derivatives with a focus on bioimaging applications.

## Author contributions

The individuals listed as authors have contributed to developing this manuscript, and no other person was involved. The authors' contributions included M.-C. R. and S. M.-H. ran the article's research and analyses and the original draft composition, and J. P. conducted the composition of the original draft, edition, conceptualization, supervision, and sources. All authors have read and agreed to the published version of this manuscript.

## Conflicts of interest

The authors declare no competing financial interest.

## Supplementary Material

RA-014-D4RA07485H-s001
