# Supplementary material for: Chemistry and properties of fluorescent pyrazole derivatives: an approach to bioimaging applications
Source: RSC Adv. 2024 Dec 11;14(53):39230–41. doi: 10.1039/d4ra07485h (PMC11632951; doi:10.1039/d4ra07485h)
Supplement: RA-014-D4RA07485H-s001 [file RA-014-D4RA07485H-s001.pdf]

Supplementary Information (SI) for RSC Advances.
This journal is © The Royal Society of Chemistry 2024
Please do not adjust margins

Journal Name

REVIEW

Chemistry and properties of fluorescent pyrazole derivatives: An approach in bioimaging applications

Santiago Melo-Hernández, María-Camila Ríos, and Jaime Portilla*

Received 00th January 20xx,
Accepted 00th January 20xx

DOI: 10.1039/x0xx00000x

www.rsc.org/

Fluorescent bioimaging, a crucial technique for in vivo studies in real cell samples, provides vital information about the metabolism of ions or molecules of biological and pharmaceutical interest. This technique typically uses probes based on organic small-molecule fluorophores, with N-heteroaromatic scaffolds playing an essential role due to their exceptional electronic and biocompatibility properties; specifically, pyrazole derivatives, with their high synthetic viability and structural diversity, are a significant and exciting option. This review presents recent (2020-2024) and prominent examples of the chemistry and properties of fluorescent pyrazole derivatives, focusing on bioimaging applications. This manuscript will inspire and motivate researchers in the field as it highlights the potential impact of your work on the future of bioimaging.

1. Introduction

In recent years, non-invasive optical methods such as fluorescent bioimaging-based ones have emerged as rapid and efficient real-time biological process monitoring in live tissue cells.[1–3] This method is vital in basic biological research and clinical use for therapeutic and diagnostic purposes, as it allows tested metabolic processes and changes in biochemical indicators and biomarkers; the obtained results are achieved using cheap and easy-to-use devices like fluorescence and confocal microscopy,[4–6] and applications include general cell staining,[7–9] labelling of subcellular structures,[10–12] and ions or small molecules sensing –*e.g.*, Cu^{2+}, reactive oxygen and nitrogen species (ROS or RNS), etc.[13–17] This technique also allows the checking of intracellular conditions like pH[18–20] and hypoxia[21–23] for sensing cancer,[24–26] bacterial infections[27–29] or ischemic injury,[30–32] and even theragnostic (therapy and diagnostics combination).[33–35] When bioimage is intended for detection, the sensing is done by changes in the fluorescence, either turn-on or turn-off upon the reaction of the probe with the analyte cells[1,2,4,5] (Fig. 1a).

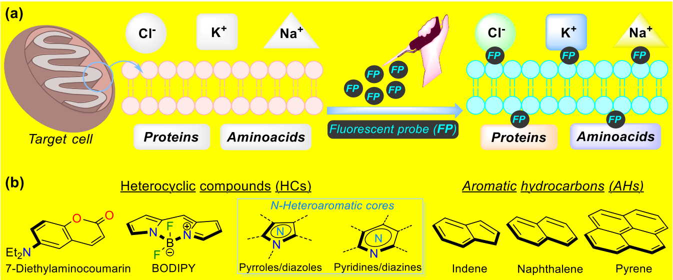

Fig. 1. a) Pictorial depiction of bioimaging acquisition by fluorescent probes. b) Structural cores of several fluorophores used for bioimaging obtention.

Considering the broad scope of applicability described above, there has been an interest in both academics and industry in developing suitable systems, platforms, and methods for taking the bioimage;[1–35] likewise, it is challenging to discover novel, highly sensible, and selective probes with good physiological compatibility.[36–38] Many fluorescent scaffolds have been used to develop new molecular probes for bioimaging applications in this area. Various of these fluorophores involve heterocyclic rings such as coumarin,[39,40] boron complexes (*e.g.*, BODIPY),[41,42] some N-heteroaromatic cores (*e.g.*, pyrroles, diazoles, pyridines, diazines, etc.);[17,43,44] however, probes based on aromatic hydrocarbons (*i.e.*, indane, naphthalene, pyrene, tetraphenylethene, among others) have also been observed despite their minor biocompatibility[34,45–47] (Fig. 1b).

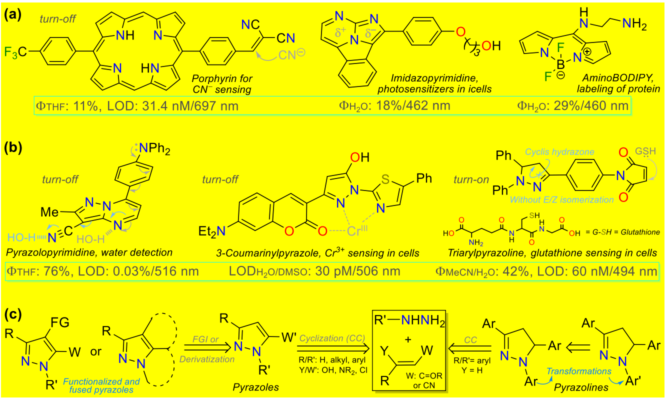

Fig. 2. a) Structure of some fluorescent N-heterocyclic dyes. b) applications in detection chemistry, and c) general syntheses of pyrazole derivatives.

Significantly, many biological and photophysical applications have been explored by using fluorophores based on N-heteroaromatic cores, as the heteroatom offers pertinent electronic properties to probes that, in addition, are usually stable compounds;[17,43,44] they also have excellent synthetic viability in the ring construction and later functionalization reactions.[43,44,48,49] Thus, exploring fluorescent N-heterocyclic compounds is a challenge in search of improving their scope and applicability, being them widely studied in the organic materials area in recent years;[43,50,51] as a result, porphyrins,[52,53] poly-pyrroles,[54] boron dipyrrolomethane (BODIPY),[55,56] and some 5:6 aza-fused diazoles with key dipolar motifs[57–59] have been successfully applied (Fig. 2a). Specifically, diverse pyrazole derivatives have shown fluorescent properties with high quantum yields and good

Bioorganic Compounds Research Group, Department of Chemistry, Universidad de los Andes, Carrera 1 No. 18A-10, Bogotá 111711, Colombia. E-mail: jportill@uniandes.edu.co

Please do not adjust margins

Please do not adjust margins

photostability, thermostability, solvatofluorochromism, etc.;[38,60–62] however, what stands out most in pyrazole derivatives is their high synthetic versatility, from the construction of the simple and fused ring to functionalization reactions of the same[43,63–65] (Fig. 2b).

Considering the bioimaging applications, pyrazole derivatives have been reported to have good membrane permeability and biocompatibility, making them suitable for bioactive and biosensing compounds, and, due to their N-donor character,[60–65] are also ideal for cation detection *in vivo*.[17,62,66] Many fluorescent pyrazoles for bioimaging purposes have been reported, with the pyrazoline ring being more frequent than the aromatic core; still, their fluorescent property is usually based on substituents or fused rings (Fig. 2b). Therefore, and as a follow-up to the last review of our group in this regard,[17] this review describes chronological reports made in the previous five years (2020-2024) of pyrazole derivatives used in bioimages. The discussion focuses on analysing five works per year, covering the compounds' syntheses and their bioimaging properties.

To better understand the purpose of this review, Table 1 presents a summary with the most pertinent results on some of the probes analysed; specifically, five examples matching each of the years explored are shown, in which the syntheses with data of applications are detailed. Table 1 and Fig. 2c show how pyrazoline, pyrazole, and fused pyrazole rings are built by cyclization reactions of 1,3-biselectrophilic reagents with hydrazine derivatives; likewise, the appropriate functionalization or derivatization of pyrazoles for the respective requests is also shown. Our primary impact on this work is the knowledge gained on the chemistry of pyrazole derivatives and their photophysical pertinency.[17,43,44,62–65] Thus, we hope that this review manuscript will be a helpful contribution to further synthetic uses in novel fluorophores synthesis for bioimaging applications.

2. Article analyses

2.1. Probes reported in 2020

First, Zhang *et al.*[72] reported the synthesis and long-term bioimaging of the pyrazoline-BODIPY hybrid probe **4** and nanoparticles (NPs) 4-NPs for ultrafast cell staining. Probe **4** was obtained in 22% yield by the Vilsmeier-Haack reaction of the *N*-phenylpyrazoline **1**, yielding the *N*-(4-formylphenyl)pyrazoline **2**; then, a one-pot reaction of **2** with 2,4-dimethylpyrrole (**3**) and BF_3 offered **4**. The probe exhibited an absorption band (λ_{abs}) at 499 nm and an emission band (λ_{em}) at 511 nm with a fluorescence quantum yield (Φ_F) of 30% attributed to an intramolecular charge transfer (ICT) from the pyrazoline ring to the BODIPY ring. The solvatofluorochromism of **4** showed a blueshift with decreased fluorescence intensity in polar solvents; however, an opposite result was observed in non-polar solvents. The rotational restriction around the BODIPY ring indicates a twisted ICT (TICT)-locally excited state effect that explains these findings. This probe showed a high Φ_F in an aqueous medium due to an aggregation-induced emission (AIE) process, and it was applied to obtain NPs that were tested on HeLa and A549 cells, finding that the fluorescence intensity had a positive dependency on the time of incubation and concentration of **4**-NPs. The *in vivo* fluorescence of the probe injected in tumours on mouse models was also explored, staining the tumour for up to 12 days, making this probe suitable for long-term, non-invasive tumour progression monitoring (Scheme 1a).

Subsequently, Wang *et al.*[67] employed probe **7** for the *in vivo* sulphite detection in the mitochondria. This probe was synthesised in 64% yield using the condensation of the *N*-(4-formylphenyl)pyrazoline with the indolinium salt **6**. The λ_{abs} at 558 nm and λ_{em} at 640 nm with Φ_F of 13% for **6** suffered visible changes in the presence of sulphite by an interruption of the ICT process due to a break in the π-conjugation upon the anion addition on the salt acting as a Michael acceptor. The observed changes were blueshift to 380 nm and 480 nm with Φ_F of 28%, finding a limit of detection (LOD) of 80 nM for sulphite. This probe was designed to locate in the mitochondria due to the interaction of the negative charge of the mitochondrial membrane and the positive charge of the indolinium salt, which serves as the guiding group; green emission was observed in the presence of sulphite, indicating that it can efficiently sense exogenous and endogenous sulphite (Scheme 1b).

Favarin *et al.*[73] obtained three gold-based pyrazoline dual probes **10a-c** in 59-64% yields by reacting chloro(triphenylphosphine)gold(I) (Ph_3PAuCl), potassium hexafluorophosphate (KPF_6), and pyrazoline-based ligands **9a-c**. These ligands exhibited absorption bands around 240 and 325 nm and emission bands around 366 nm, and although they are fluorescent dyes, gold complexes enhanced the emission via charge transfer (CT) processes governed by the metal-to-ligand CT (MLCT). The MLCT favours the intraligand CT (ILCT) and ligand-to-ligand CT (LLCT), as shown by DFT calculations in the ground state, leading the authors to assume that the emission of complexes **10a-c** is due to the mentioned CT processes. However, the photophysical mechanism in the excited state needs to be studied in more depth as they are less simple. These CT processes led to two λ_{em}, a blue one when excited at about 305 nm and a green one when excited at 405nm. The cellular imaging was performed on two cancer cell lines (MDA-MD231 and MCF-7) and one non-cancerous healthy cell line (HUVEC). For studied cell lines, **10a-c** showed good internalisation and staining of the cytoplasm and seem to be suitable dyes for general staining or even for co-staining assays as its dual emission may allow using further dyes that are compatible with at least one of the fluorescence modes of **10a-c**, increasing their span of utility. However, it must be noted that **10a-c** resulted in highly cytotoxic for cancer and non-cancer cell lines, which would impair their application for *in vivo* imaging as their use would prevent studying cellular processes and lead to the death of the cells (Scheme 1c).

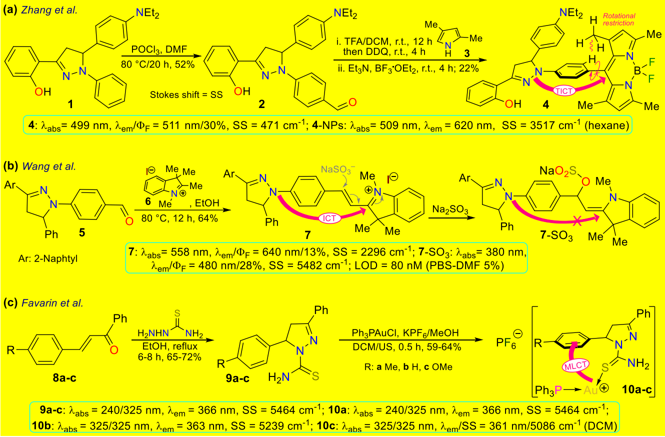

Scheme 1. Synthesis and optical data of pyrazolines a) **4**, b) **7**, and c) **10a-c**.

Alizadeh *et al.*[74] used the pyrazolopyridine-coumarin hybrid probes **13a-h** for general cell staining, synthesised in 70-85% yield via 1,3-dipolar cycloaddition reaction of hydrazonoyl chloride **11** and the enone **12**. The reaction proceeded in the presence of ammonium acetate by nucleophilic substituting the chloride on the coumarin

Please do not adjust margins

moiety with the nitrogen source, forming an enamine that then reacted with the carbonyl to generate products after aromatization. These probes exhibited coplanar, rigid, and aromatic structures responsible for their fluorescence, except probes bearing a nitro group as it favours the internal conversion over radiative decay; likewise, the emission spectra were barely affected by the substituents. As a representative example, probe **13a** exhibited λ_{abs} at 330 nm, λ_{em} at 465 nm, Φ_F of 65%, good photostability, and pH sensitivity; as a result, this probe was taken for cellular imaging of MG-63 cells. The probe showed good internalisation into the cell, apparently even into the nuclei, as there were no black spots on the images after 30 minutes of incubation (Scheme 2a).

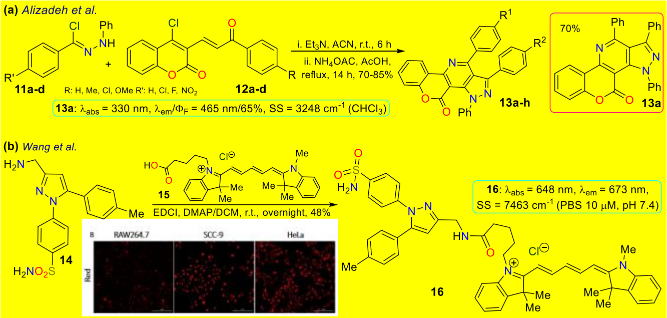

Scheme 2. Synthesis and photophysical data of a) the fused pyrazoles **13a-h** and b) the π-extended pyrazole **16**. Bioimage of **16** in RAW 264.7, SCC-9 and HeLa cell lines by ref.[75] with minor modifications licensed under Creative Commons CC BY 4.0.

As a final example in 2020, Wang *et al.*[75] obtained the NIR fluorescent probe celecoxib-based **16** in 48% yield by the amidation reaction of the 3-aminomethylpyrazole **14** with the carboxylic acid **15** bearing a cyanine moiety; this probe was used in cyclooxygenase sensing 2 (COX-2) activity in tumoral cells. The photophysical properties of probe **16** were recorded in PBS at a pH of 7.4 to achieve conditions as close as possible to the cellular environment, displaying λ_{abs} at 648 nm and λ_{em} at 673 nm; in addition, docking studies showed that **16** is a potent inhibitor of COX-2 whose binding site is the same as free celecoxib, *i.e.*, inside the active side pocket. The COX-2 inhibiting activity of **16** was confirmed by *in vitro* tests using purified COX-2m, and its actual capability in living cells was performed by imaging assays in a normal cell line (RAW264.7) and two cancer cell lines (HeLa and SCC-9). Very low toxicity to the three cell lines with differential and scarce fluorescence for normal cells but strong fluorescence for cancer cells was evidenced; thus, **16** could discern normal and cancerous cells. As a result, the optical mechanism for **16** must be associated with its accumulation around COX-2, which is less expressed in normal cells, explaining why RAW264.7 cells barely shined while cancer cells strongly fluoresced (Scheme 2b).

2.2. Probes reported in 2021

First, He *et al.*[76] synthesised the (pyrazolin-3-yl) phenyl acrylate **19a-b** in 50-60% yield by the esterification between the 2-(pyrazolin-3-yl)phenol **17a-b** and acryloyl chloride **18**. These probes exhibited very weak emission at 490 nm, but it increased upon the nucleophilic addition of glutathione to the acrylate receptor fragment **19a-b**. By DFT studies, the authors observed that the high occupied molecular orbital (HOMO) is focused on the *N*-phenylpyrazoline moiety and the low unoccupied molecular orbital (LUMO) on the acrylate, causing an ICT with a fluorescence turn-off; thus, the LUMO is disturbed upon the conjugated addition of the respective thiol group, raising the energy gap HOMO-LUMO and thereby preventing the quenching. The probes offered low toxicity, and **19a** was taken for imaging tests in cells cultured with and without glutathione; the incubation of the cells with **19a** led to blue fluorescence inside the cells without any morphological artifice thereof, indicating that **19a** can detect endogenous glutathione as a new helpful tool for studying glutathione's biological role in different processes (Scheme 3a).

Huang *et al.*[77] obtained the coumarin-3-ylpyrazoline **24** for *in vivo* hypochlorite sensing by reacting the 3-acetylcoumarin **20** with *p*-tolualdehyde (**21a**) to produce the enone **22** that then reacts with the arylhydrazine **23** to afford **24** in 49% yield. Probe **24** shows λ_{abs} at 375 nm, λ_{em} at 520 nm, and a donor-acceptor structure from an electron-donating group (EDG), such as diethylamino, to an electron-withdrawing group (EWG) hydrazone in the pyrazoline fragment, favouring the emission of **24** by an ICT phenomenon. This probe was degraded to the dicarboxylic acid **25** through hypochlorite-mediated oxidation upon adding the analyte (on the coumarin ring), quenching its fluorescence. The fluorophore **24** showed slight cytotoxicity and good internalisation into the cytoplasm for cell imaging. When the cells or the zebrafish were cultured without analyte, the probe showed a strong green fluorescence, quenched upon the analyte addition, implying that **24** can detect exogenous ClO^-. HeLa cells were also incubated with LPS to stimulate the endogenous production of ClO^-, which was also detected (Scheme 3b).

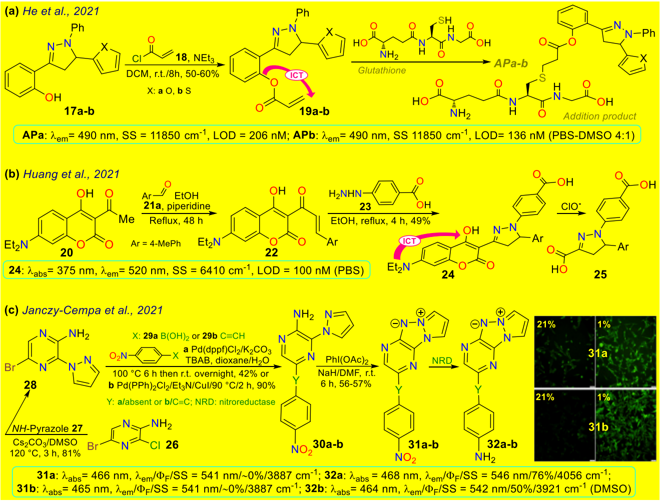

Scheme 3. Synthesis and photophysical data of a) **19a-b**, b) **24**, and c) **31/32a-b**. Bioimaging of A2058 cells cultured under normoxic (21% pO_2) and hypoxic (1% pO_2) conditions 24 h with **31a-b** (prior 2 h at 8 µM); ref.[68] with minor changes licensed under Creative Commons CC BY 4.0.

Janczy-Cempa *et al.*[68] used the nitrocompounds **31a-b** as probes sensitive to nitroreductase, allowing *in vivo* hypoxia evaluation by imaging. Probes were synthesised by reacting the pyrazine **26** with *NH*-pyrazole (**27**) to form the *N*-hetarylpyrazole **28** that then reacted with the appropriate nitroaryl derivative **29a-b** to yield intermediates **30a-b** that finally were subject to a PIDA-mediated cyclization reaction (Scheme 3c). Probes **31a-b** show λ_{abs} around 465 nm, λ_{em} around 541 nm, and very weak fluorescence as the nitro group quench the process. The reduced fluorophores **32a-b** showed good photostability and responses against human nitroreductase (NRD), even with interferents, and their fluorescence was not affected by molecular oxygen. To evaluate the bioimaging suitability of probes, they were cultured with A2058 cells under normoxic and hypoxic conditions. Cells grown under normoxic conditions showed a slight fluorescence emission, possibly due to the activity of basal levels of NRD. However, when subjected to hypoxic conditions (less than 1%

Please do not adjust margins

oxygen), the cells showed increased green emission, indicating a more significant reduction of probes **31a-b**.

Krishnaveni *et al.* [78] reported the bisimine **35** as a probe for *in vivo* detection of Zn^{2+} that was prepared by the condensation reaction of 5-bromosalicylaldehyde (**21b**) with hydrazine and then with the 4-formylpyrazole **34** to produce the probe in 98% yield. Probe **35** exhibited two λ_{abs} at 288 and 366 nm, a weak λ_{em} at 511 nm, a HOMO spreading over the entire molecule, and a LUMO focused over the imine-bromophenol moiety. The no fluorescence for **35** is due to the *s-cis/s-trans* isomerization of the bisimine group (–CH=N–N=CH–). However, when **35** complexed with Cd^{2+} or Zn^{2+}, the HOMO focused on the metal, and LUMO remains similar; thus, the fluorescence in **35**-M is attributed to a metal-to-ligand charge transfer (MLCT). This probe detected Zn^{2+} in HeLa cells and zebrafish embryos, exhibiting little toxicity. Zebrafish do not fluoresce with **35** and without the analyte, but an intense yellow emission was observed under the exposition and internalisation with Zn^{2+} (Scheme 4a).

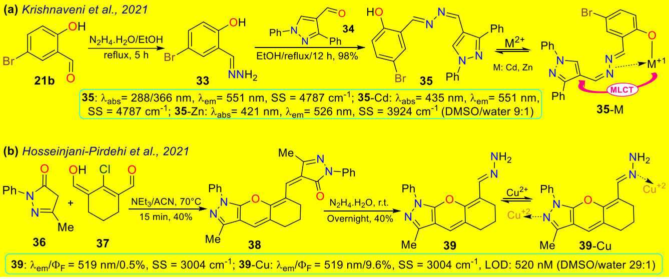

Scheme 4. Synthesis and photophysical data of a) **35**/**35**-M and b) **39**/**39**-Cu.

In the last example of 2021, Hosseinjani-Pirdehi *et al.*[79] obtained the tetrahydro-chromeno[2,3-*c*]pyrazole **39** by reacting 2 equivalents of the pyrazolone **36** with the formylated 1,3-biselectrophyle **37** to produce **38** that ultimately suffered hydrazinolysis at **39** in 40% yield (Scheme 4b). This probe was used for *in vivo* Cu^{2+} sensing and both **39** and **39**-Cu showed no displacements on the λ_{abs} and λ_{em}, but the fluorescence was enhanced in **39**-Cu. For **39**, no fluorescence was observed due to the *E/Z* isomerization of the hydrazone moiety, which is restricted once the metal chelated, causing more rigidity on the molecule. This probe was used for bioimaging and *in vivo* detection of Cu^{2+}; cells incubated solely with **39** showed no emission. However, the cells showed a greenish-yellow emission after Cu^{2+} was added to the culture. This result indicates that **39** has reasonable internalisation rates and can detect intracellular Cu^{2+}; however, it is essential to note that **39** showed significant toxicity towards cells. Given this, **39** might not be the best tool for ion monitoring in living systems, but it could still be used for *in vitro* or *ex vivo* testing.

2.3. Probes reported in 2022

Liang *et al.*[37] obtained 1,4-disubstituted pyrazoles **44** and **48** for *in vivo* detection of c-Met, a protein highly expressed in solid tumours that is a strategic target for cancer diagnosis and detection. The synthesis started from the arylaldehyde **21c** or anhydride **45, and it continued** incorporating a bromo-alkyl moiety that was then stirred with the pyrazolyl-boronic acid **41** to form boronic esters **42** and **47**, which were subjected to a Suzuki-Miyaura reaction yielding **44 and 48**. Both dyes show a photoinduced electron transfer (PET) process with fluorescence turn-off, in which the excited coumarin moiety transfers an electron to the receptor quinoline moiety; however, this process is inhibited upon binding to c-Met, turning on the emission of **44** and **48** (Scheme 5a and 5b). As for the bioimaging assays, both probes exhibited slight fluorescence in normal cells, which related to basal levels of c-Met as it is a protein linked to several metabolic processes for almost any cell. In contrast, when the fluorophore was used for a c-Met overexpressing cell line, there was an apparent increase in the fluorescence, indicating that both probes can be used for detecting changes in the levels of c-Met. However, it must be considered that both dyes were highly toxic to at least two cell lines; thus, its application spectrum might not be as broad as expected.

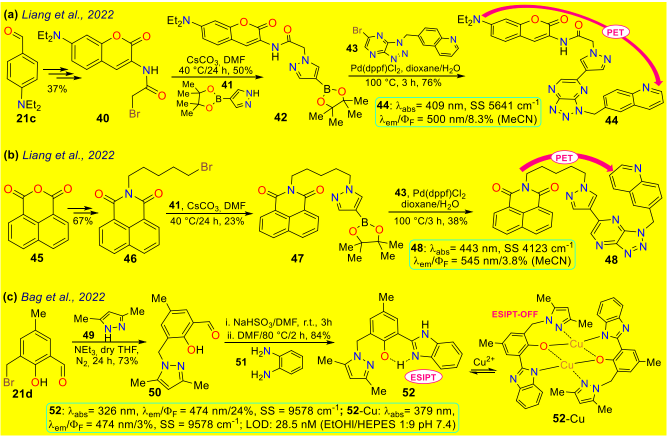

Scheme 5. Synthesis and photophysical data of a) **44**, b) **48**, and d) **52**/**52**-Cu.

Later, Bag *et al.*[80] synthesised pyrazole derivative **52**, an excited-state intramolecular proton-transfer (ESIPT) active sensor for sensing Cu^{2+} in cells and plants, starting from the 3-bromomethylsalicylaldehyde **21d** and the *NH*-pyrazole **49** to form the intermediate **50**; then, **50** reacted with sodium bisulphite and later with *o*-phenylenediamine **51** to yield the benzimidazole derivative **52.**[80] Unlike other sensors for metal cations analysed above, **52** is a turn-off sensor whose emission was quenched by adding Cu^{2+}; this dye exhibited a λ_{abs} at 326 nm and a λ_{em} at 474 nm with Φ_F of 24%, whereas **52-Cu** showed a λ_{abs} redshifted at 379 nm with a λ_{em} no shifts but dramatically decreased (Φ_F = 3%) due to an ESIPT. In the excited state, the OH group of **52** forms intramolecular hydrogen bonds with pyridine-like nitrogen in pyrazole and benzimidazole rings, giving a strong blue emission; still, the fluorescence is quenched upon the formation of **52-Cu** as the heteroatoms are occupied. About the use of **52** for *in vivo* Cu^{2+} sensing, MCF-7 cells, chickpeas, and Mung beans were tested. For cells, **52** showed almost no toxicity; samples treated with **52** without Cu^{2+} showed strong blue emission inside the cells and the sprout of the beans, revealing that **52** is well absorbed in mammal and plant cells. In contrast, when the samples were treated with **52** and Cu^{2+}, fluorescence was quenched in both cells and plants.

Roy *et al.*[69] obtained the pyrazolone **54** by reacting 3-methoxy-salicylaldehyde (**21e**) with the aminopyrazolone **53**. This probe showed three λ_{abs} at 230, 268, and 326 nm and was not fluorescent due to PET and ESIPT processes, but the complex **54**-Al redshifted to 404 nm; upon excitation of **54**, its aryloxyimine group transfers one electron to the adjacent enone moiety, and the molecular conformation allows the intramolecular hydrogen bond formation. Notably, PET and ESIPT processes are not viable after forming **54**-Al as heteroatom donors are arrested in chelating the Al^{3+} (Scheme 6a); likewise, **54** was used for intracellular Al^{3+} sensing by bioimaging, observing an absence of emission for cells treated with Al^{3+}, but once the dye was added, a strong green fluorescence was seen. Nevertheless, care must be taken when using this probe as it was confirmed with an acridine orange/ethidium bromide co-staining

Please do not adjust margins

Please do not adjust margins

assay that the **54**-Al complex may interact with single-stranded DNA, affecting the cell cycle and leading to cell death via apoptosis.

Finally, Kuznetsov *et al.*[81] prepared the phosphorescent probe **59** by the Suzuki coupling of the arylboronic acid **55** with the heteroaryl chloride **56**, yielding the tridentate ligand **57** that then reacted with the Eu(III) complex **58**, delivering **59**. This probe showed two λ_{abs} at 344 and 391 nm with a λ_{em} at 612 nm, typical for Eu(III) complexes (Scheme 6b); as NIR radiation is minor phototoxic and more penetrating than visible radiation, these results offer a probe that does not allow cellular photodamage but has deep tissue penetration. By testing the temperature effect on the luminescence of **59**, it was found that raising the temperature reduces the lifetime of its emission, indicating its high sensitivity to temperature changes. Nanoparticles **59**-NPs with a superficial positive charge were also prepared as their target is the mitochondrion, whose membrane is anionic; still, **59**-NPs were found in endosomes, lysosomes or similar parts, not in the mitochondria. Moreover, phosphorescence lifetime imaging microscopy (PLIM) assays were used by uncharged **59**-NPs for sensing cellular temperature; the images show a general typical temperature in cells, and some colder spots can be seen. However, these results are good enough to indicate that the probe is suitable and viable for intracellular temperature review. The same behaviour was found for modified **59**-NPs, indicating that the cationic surface does not affect its sensing properties.

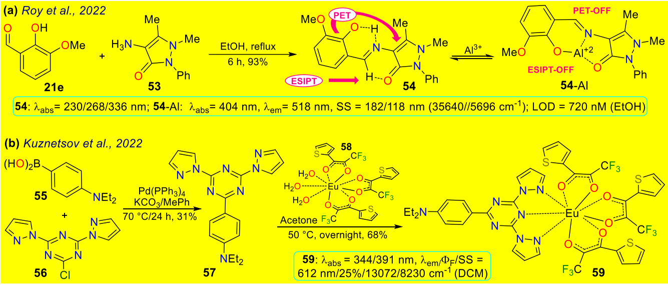

Scheme 6. Synthesis and photophysical data of probes a) **54**/**54**-Al and b) **59**.

2.4. Probes reported in 2023

Krishnan *et al.*[82] synthesised the pyrazolo[4,3-*b*]pyridine **62** in 85% yield through the Povarouv reaction of the 5-aminopyrazole **60** with the arylaldehyde **61** and used this probe for the detection of BF_3 in E. Coli and HeLa cells. Probe **62** displayed an absorption band at 336 nm and an emission band at 440 nm, which were then redshifted in the presence of BF_3 –at 368 and 473 nm, respectively. Upon the gradual BF_3 addition, the absorption band of probe **62** decreased while the fluorescence band increased, increasing the quantum yield from 35 to 65% due to an ICT from the alkoxy group to the boron atom. Probe **62** was tested in the presence of E. Coli cells, observing a blue fluorescence once the bacterial culture was incubated with BF_3; 3-(4,5-dimethylthiazol-2-yl)-2,5-diphenyltetrazolium bromide (MTT) assays were also made, offering a green fluorescence, which indicates that the intracellular uptake of BF_3 resulted in the complexation in the intracellular region (Scheme 7a).

In another work, Shi *et al.*[70] prepared the 3-(coumarin-3-yl)pyrazole **65** by two steps in 87% yield, implying a cyclocondensation reaction of the β-diketone **63** with hydrazine, followed by a sulfonylation reaction with 2,4-dinitrobenzenesulfonyl chloride (**64**). This probe exhibited a λ_{abs} at 425 nm and a λ_{em} at 500 nm without a shift on these wavelengths in the presence of H_2S; nevertheless, upon the analyte addition, the solution changed its colour, and the Φ_F enhanced from 5% to 27% as a PET-OFF process is activated once the sulfonyl group is cleaved in **64** by the H_2S. Competitivity assays were performed with varied cations, anions, and some amino acids, finding that the reaction with H_2S was unperturbed by the presence of other analytes and lasted 3 min; still, with other analytes like cysteine, the reaction took 30 minutes or longer. Ultimately, the authors investigated the recognition of exogenous and endogenous H_2S in MCF-7 cells, which were incubated with the analyte and a fluorescence turn-on on the cells with LOD of 18.7 nM (Scheme 7b).

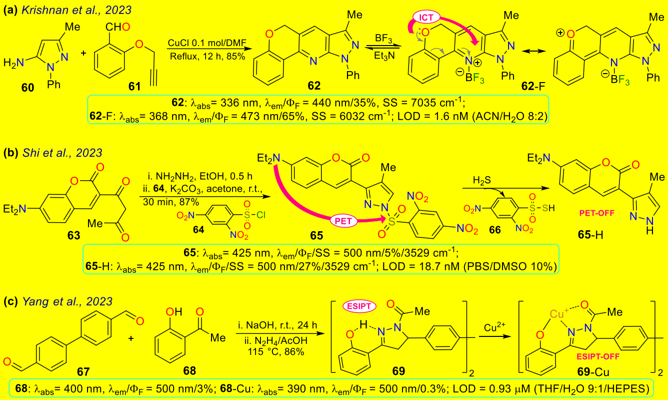

Scheme 7. Synthesis and optical data of probes based on a) pyrazolopyridine **62**/**62**-F, b) 3-(coumarin-3-yl)pyrazole **65**/**65**-H, and c) pyrazoline **69**/**69**-Cu.

Yang *et al.*[83] synthesised the bispyrazoline **69** in 86% yield through the condensation reaction of 4,4'-diformylbiphenyl (**67**) with 2-hydroxyacetophenone **68** followed by cyclization reaction with hydrazine. Probe **69** exhibited a λ_{abs} at 400 nm, shifted to 390 nm in the presence of Cu^{2+}, and a λ_{em} at 500 nm. In the presence of the analyte, the fluorescence decreased with Φ_F from 3% to 0.3% due to an interrupted ESIPT process. Biological assays were performed on HeLa cells with **69** and **69**-Cu, evidencing that these cells had a blue emission with good permeability in the presence of **69**, and by adding Cu^{2+} to the system, the fluorescence signal weakened, making it suitable for the detection of intracellular copper ions (Scheme 7c).

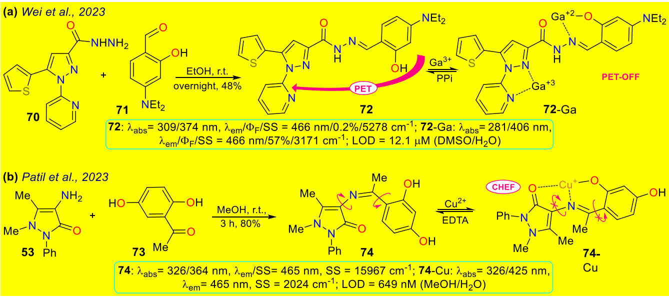

Scheme 8. Synthesis and optical data of a) **72**/**72**-Ga and b) **74**/**74**-Cu.

Two examples of pyrazoles reported in 2023 that do not present an approach to bioimaging but focus on the design of fluorophores are discussed below. Firstly, Wei *et al.*[84] obtained **72** by condensing the 3-pyrazolylhydrazide **70** with the salicylaldehyde **71**. This probe showed two λ_{abs} at 309 and 374 nm shifted to 281 and 406 nm upon the addition of Ga^{3+} ions; likewise, weak fluorescence was observed (Φ_F = 0.2%) that increased (Φ_F = 57%) in the presence of the analyte obtaining a LOD of 12.1 mM. The almost no fluorescence of **72** is due to a PET-OFF process in the complex **72**-Ga. The authors studied the effect of the diethylamino group in **72**, evidencing the necessity of this group for the photophysical properties (Scheme 8a). Second, Patil *et al.*[85] synthesised the imine **74** in 80% yield using the reaction

Please do not adjust margins

Please do not adjust margins

of 4-aminoantipyrine (**72**) with 2,5-dihydroxyacetophenone (**73**); this dye exhibits two λ_{abs} at 326 and 364 nm, but in the presence of Cu^{2+}, the band at 364 nm shifted to 425 nm (Scheme 8b). Upon increasing the concentration of Cu^{2+}, a progressive rise and decrease of peaks at 425 and 364 nm, respectively, was evidenced due to the formation of the complex **74**-Cu, which favours a ligand-to-metal charge transfer (LMCT). An emission peak at 465 nm was evidenced for **74** and **74**-Cu with a fluorescence enhancement in the presence of Cu^{2+} due to a chelation-enhanced fluorescence process (CHEF).

2.5. Probes reported in 2024

Rasin *et al.*[71] synthesised the fused pyrazole **78** by the cyclization reaction of the 1,3-biselectrophyle **76** with the thiosemicarbazide **77**. Probe **78** exhibited two absorption bands at 325 nm and 372 nm, and upon the addition of fluoride ions, a new band emerged around 430 nm, indicating the formation of **78**-F; **78** has a λ_{em} at 476 nm that is shifted to 492 nm for **78**-F with an increase in the Φ_F from 38% to 64%. Complex **78**-F has a fluorescence enhancement compared to **78**, which links the relaxation from the stimulated state to the PET process. The authors investigated the detection of the anion on T24 living cells, and a blue fluorescence was observed once the cells were treated with the sensor and fluoride ions, indicating that **78** could detect the anion on the cell's cytoplasm.

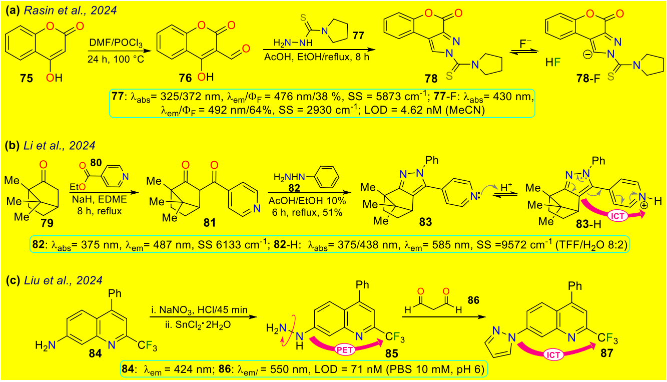

Scheme 9. Synthesis and photophysical data of fluorophores a) **78**/**78**-F, b) **83**/**83**-H, and c) **85**/**87**.

Li *et al.*[86] designed the probe **83** to detect aqueous acid, which was obtained using the condensation reaction of the bicyclic ketone **79** with ethyl isonicotinate **80** to form the β-diketone **81** that finally cyclocondensed with phenylhydrazine (**82**). Probe **83** exhibits an absorption band at 375 nm, which gradually diminished with the medium acidity increase, and a new peak emerged at 438 nm; likewise, an emission band at 487 nm was showed and decreased as the acidity increased, but a new peak at 585 nm emerged; these spectral changes are caused by the protonation of the pyridine ring generating an ICT effect that leads to the redshift of the spectrum. The authors conducted fluorescent bioimage studies indicating that **83** exhibited low cytotoxicity, making the probe well suited for monitoring pH alterations of zebra fishes as they show a strong fluorescence on acidic media (Scheme 9b).

After, Liu *et al.*[87] obtained the pyrazole **87** by the cyclocondensation reaction of malonaldehyde (**86**) with the hetarylhydrazine **85**, which was obtained via diazonium salts from the hetarylamines **84** (Scheme 9c). The 4-trifluoromethylquinoline group of **85** is the Golgi-target, and the hydrazine group is the recognition site; this probe exhibits an emission band at 424 nm but has no fluorescence due to a PET process generated by the presence of the amino group. However, once the probe reacts with **86,** forming **87**, the electron donating capacity of the amino group is affected, offering both PET-OFF and ICT phenomena with a redshift in the emission spectra and an enhanced fluorescence. The MTT method was used to **87**, and low cytotoxicity was found. Then, the ability of **85** to Golgi apparatus was proved by labelling HeLa cells and red staining the Golgi apparatus, detecting a blue emission, indicating the excellent targeting of the probe with the desired target with a LOD of 71 nM.

Deng and colleagues prepared probe **89** using the reaction of the tetrazine **88** with phenyl-propanenitrile, a type of [4+1] cycloaddition that is an alternative to the inverse electron-demand Diels-Alder reaction that has gained attention in protein labelling and drug delivery thanks to biocompatibility, rapid kinetics and payload release (Scheme 10a).[88] Probe **89** exhibits absorption and emission bands at 394 and 551 nm, respectively, with a Φ_F of 42% on PBS buffer, probably given an ICT-OFF process that initially is from the dialkylamino group to the π-deficient tetrazine ring. This probe was studied using live 193T-cell labelling on intracellular targets (nucleus proteins and mitochondria), finding vivid mitochondria stains and orthogonal labelling in the membrane, making probe **89** suitable for bioimage labelling.

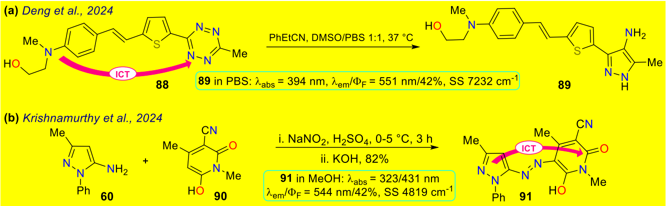

Scheme 10. Synthesis and photophysical data of probes a) **89** and b) **91**.

Finally, Krishnamurthy *et al.*[89] synthesised probe **91** in 82% yield starting from the 5-aminopyrazole **60** that undergoes diazotization followed by a coupling reaction with the cyanomethyl-pyridone **90**. Probe **91** exhibited two absorption peaks at 323 nm and 431 nm and an emission peak at 544 nm with a Φ_F of 42% in methanol, which helps stabilise the hydrazo tautomer **91**; in solvents such as MeOH, DMSO, and DMF, the hydrazo form is stabilised generating a redshift on the absorption peaks while in MeCN, DCM and chloroform the azo form is stabilised generating a blueshift on the spectra. An ICT process is responsible for the fluorescence of probe **91**; this property was used for bioimaging in HeLa cells as a strong green fluorescence was observed and studied for the electrochemical detection of dopamine with LOD of 0.81 mM (Scheme 10b).

3. Conclusions

In summary, diverse synthetic transformations implying fluorescent pyrazoles have been reported, some by the ring construction and others using functionalization or derivatization strategies, usually by classical reactions and reaction conditions easily reproducible; in this manner, novel functional pyrazoles with relevant applications have been obtained finding some examples in bioimaging applications. These fluorescent dyes have proven to have exceptional results in general cell staining and the detection of in-cell conditions like temperature or hypoxia. The compounds herein discussed include further structural motifs, from additional heterocyclic, pyrazolines, or fluorescent groups to complexes with metal ions, for which different synthetic approaches are needed. For the evaluated systems, ICT, PET, and MLCT are the most prevailing photophysical phenomena that govern the fluorescence of the probes, which, upon

This journal is © The Royal Society of Chemistry 20xx

Please do not adjust margins

Please do not adjust margins

reacting with the target (like metal ions, small molecules, or enzymes), are either enhanced or 'turned-off', leading to observable changes in the fluorescence that allow the sensing of the different targets, which in turn is what leads to pyrazole derivatives to have so many applications as somewhat. Various molecular architectures pyrazoles-based have been used in bioimaging applications, in which the incorporation of EDGs or highly conjugated substituents is the main constant in the probe design; probes must be functionalized with appropriate receptors (*e.g.*, ion receptors, lipophilic fragments, or hydrogen bond mediators) so that they can perform their work in different environments. Notably, although there are many published articles on fluorescent probes for bioimaging applications based on small molecules, several of these works do not carry out an appropriate photophysical study for the respective development; as a result, a good look at this review could pointedly improve this issue.

Data availability

This review manuscript presents an original work submitted for publication only in RSC Advances. The authors have no conflicts of interest to report with this submission, and they have seen, revised, and approved this paper, which details the chemistry and properties of fluorescent probes based on pyrazole/pyrazoline derivatives with an approach regarding bioimaging applications.

Conflicts of interest

The authors declare no competing financial interest.

Authors Contributions

The individuals listed as authors have contributed to developing this manuscript, and no other person was involved. The authors' contributions included M.-C. R. and S. M.-H. ran the article's research and analyses and the original draft composition, and J. P. conducted the composition of the original draft, edition, conceptualization, supervision, and sources. All authors have read and agreed to the published version of this manuscript.

Biography of authors

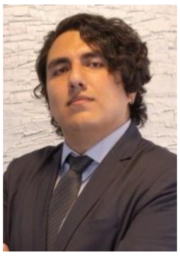

Santiago Melo-Hernandez was born in Bogotá (Colombia) and received his BSc Degrees in Chemistry (2020) and Microbiology (2021), both at Universidad de Los Andes (Bogotá). Currently, he works as a patent advisor at a law firm, being in close contact with the latest developments in different industries, especially in the pharmaceutical and biotechnology fields. His research interest focuses on synthesising compounds with potential biological activity and biological evaluation to develop new therapeutic and/or diagnostic leads.

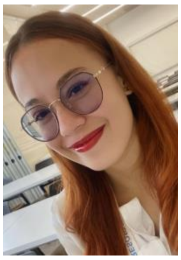

Maria-Camila Rios was born in Medellin (Colombia); she received his BSc Degree in 2021 and her MSc in 2024 at the Universidad de Los Andes (Bogotá). Currently, she works as a research assistant at the bioorganic compounds research group in the same institution under the supervision of Prof Jaime Portilla. Her research interest focuses on synthesising heterocyclic compounds with biological or photophysical relevance.

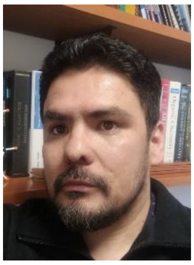

Jaime Portilla was born in Cali (Colombia); he is a research professor at the Department of Chemistry of the Universidad de Los Andes in Bogotá, and he led the bioorganic compounds research group in 2008. He has completed his PhD in Organic Synthesis under the supervision of Prof. J. Quiroga (2007) at the Universidad del Valle (Cali). His current research interests involve the areas of supramolecular chemistry and molecular recognition. Portilla's group research focuses on eco-compatible organic synthesis approaches, predominantly in 5:6 aza-fused heterocyclic compounds of biological or photophysical potential.

Acknowledgments

The authors thank the Chemistry Department and Vicerrectoría de Investigaciones from Universidad de los Andes for financial support. JP acknowledged support from the science faculty (project INV-2023-162-2810). JP also wishes to credit all Bioorganic Compounds Research Group members for their valuable collaboration in syntheses and properties of pyrazole derivatives.

Notes and references

1 Z. Guo, S. Park, J. Yoon and I. Shin, Recent progress in the development of near-infrared fluorescent probes for bioimaging applications, *Chem. Soc. Rev.*, 2014, **43**, 16–29.

2 M. Won, M. Li, H. S. Kim, P. Liu, S. Koo, S. Son, J. H. Seo and J. S. Kim, Visible to mid IR: A library of multispectral diagnostic imaging, *Coord. Chem. Rev.*, 2021, **426**, 213608.

3 T. Terai and T. Nagano, Fluorescent probes for bioimaging applications, *Curr. Opin. Cell Biol.*, 2008, **12**, 515–521.

4 T. Terai and T. Nagano, Small-molecule fluorophores and fluorescent probes for bioimaging, *Pflügers Arch.*, 2013, **465**, 347–359.

5 B. A. D. Neto, J. E. P. Sorto, A. A. M. Lapis and F. Machado, Functional chromophores synthesized via multicomponent Reactions: A review on their use as cell-imaging probes, *Methods*, 2023, **220**, 142–157.

6 J. Milczarek, R. Pawlowska, R. Zurawinski, B. Lukasik, L. E. Garner and A. Chworos, Fluorescence and confocal imaging of mammalian cells using conjugated oligoelectrolytes with phenylenevinylene core, *J. Photochem. Photobiol. B*, 2017, **170**, 40–48.

Please do not adjust margins

Please do not adjust margins

7 V. P. De Souza, V. Vendrusculo, A. M. Morás, L. Steffens, F. S. Santos, D. J. Moura, F. S. Rodembusch and D. Russowsky, Synthesis and photophysical study of new fluorescent proton transfer dihydropyrimidinone hybrids as potential candidates for molecular probes, *New J. Chem.*, 2017, **41**, 15305–15311.

8 Y. Li, Q. Chen, X. Pan, W. Lu and J. Zhang, Development and Challenge of Fluorescent Probes for Bioimaging Applications: From Visualization to Diagnosis, *Top. Curr. Chem.*, 2022, **380**, 22.

9 D. Pirone, J. Lim, F. Merola, L. Miccio, M. Mugnano, V. Bianco, F. Cimmino, F. Visconte, A. Montella, M. Capasso, A. Iolascon, P. Memmolo, D. Psaltis and P. Ferraro, Stain-free identification of cell nuclei using tomographic phase microscopy in flow cytometry, *Nat. Photonics*, 2022, **16**, 851–859.

10 Mayank, R. Rani, A. Singh, N. Garg, N. Kaur and N. Singh, Mitochondria- and nucleolus-targeted copper(i) complexes with pyrazole-linked triphenylphosphine moieties for live cell imaging, *Analyst*, 2020, **145**, 83–90.

11 M. Gao, H. Su, S. Li, Y. Lin, X. Ling, A. Qin and B. Z. Tang, An easily accessible aggregation-induced emission probe for lipid droplet-specific imaging and movement tracking, *Chem. Commun.*, 2017, **53**, 921–924.

12 A. Reicher, J. Reiniš, M. Ciobanu, P. Růžička, M. Malik, M. Siklos, V. Kartysh, T. Tomek, A. Koren, A. F. Rendeiro and S. Kubicek, Pooled multicolour tagging for visualizing subcellular protein dynamics, *Nat. Cell Biol.*, 2024, **26**, 745–756.

13 M. O. Rodrigues, M. N. Eberlin and B. A. D. Neto, How and Why to Investigate Multicomponent Reactions Mechanisms? A Critical Review, *Chem. Rec.*, 2021, **21**, 2762–2781.

14 S. Das, H. K. Indurthi, P. Asati and D. K. Sharma, Small Molecule Fluorescent Probes for Sensing and Bioimaging of Nitroreductase, *ChemistrySelect*, 2022, **7**, e202102895.

15 J. T. Hou, K. K. Yu, K. Sunwoo, W. Y. Kim, S. Koo, J. Wang, W. X. Ren, S. Wang, X. Q. Yu and J. S. Kim, Fluorescent Imaging of Reactive Oxygen and Nitrogen Species Associated with Pathophysiological Processes, *Chem*, 2020, **6**, 832–866.

16 L. C. Murfin, M. Weber, S. J. Park, W. T. Kim, C. M. Lopez-Alled, C. L. McMullin, F. Pradaux-Caggiano, C. L. Lyall, G. Kociok-Köhn, J. Wenk, S. D. Bull, J. Yoon, H. M. Kim, T. D. James and S. E. Lewis, Azulene-Derived Fluorescent Probe for Bioimaging: Detection of Reactive Oxygen and Nitrogen Species by Two-Photon Microscopy, *J. Am. Chem. Soc.*, 2019, **141**, 19389–19396.

17 A. Tigreros and J. Portilla, Fluorescent Pyrazole Derivatives: An Attractive Scaffold for Biological Imaging Applications, *Curr. Chin. Sci.*, 2021, **1**, 197–206.

18 H. Chen, F. Ding, Z. Zhou, X. He and J. Shen, FRET-based sensor for visualizing pH variation with colorimetric/ratiometric strategy and application for bioimaging in living cells, bacteria and zebrafish, *Analyst*, 2020, **145**, 4283–4294.

19 S. Gond, P. Yadav, B. S. Chauhan, S. Srikrishna and V. P. Singh, Development of an 'OFF-ON-OFF' colorimetric and fluorometric pH sensor for the study of physiological pH and its bioimaging application, *J. Mol. Struct.*, 2022, **1252**, 132147.

20 X.-L. Xue, Y. Wang, S. Chen, K.-P. Wang, S.-Y. Niu, Q.-S. Zong, Y. Jiang and Z.-Q. Hu, Monitoring intracellular pH using a hemicyanine-based ratiometric fluorescent probe, *Spectrochim. Acta - A: Mol. Biomol.*, 2023, **284**, 121778.

21 N. Bauer, I. Maisuls, A. Pereira da Graça, D. Reinhardt, R. Erapaneedi, N. Kirschnick, M. Schäfers, C. Grashoff, K. Landfester, D. Vestweber, C. A. Strassert and F. Kiefer, Genetically encoded dual fluorophore reporters for graded oxygen-sensing in light microscopy, *Biosens. Bioelectron.*, 2023, **221**, 114917.

22 V. Mirabello, F. Cortezon-Tamarit and S. I. Pascu, Oxygen Sensing, Hypoxia Tracing and in Vivo Imaging with Functional Metalloprobes for the Early Detection of Non-communicable Diseases, *Front. Chem.*, 2018, **6**, 00027.

23 D. Chen, W. Wang, Q. Zhu, Q. Wang, D. Quan, Y. Zeng, K. Li, Y. Zhou, C. Liu, W. Zhan and Y. Zhan, In vivo real-time monitoring of the development of hypoxia and angiogenesis in cervical cancer, *J. Chem. Eng.*, 2023, **473**, 145498.

24 P. Feng, H. Zhang, Q. Deng, W. Liu, L. Yang, G. Li, G. Chen, L. Du, B. Ke and M. Li, Real-Time Bioluminescence Imaging of Nitroreductase in Mouse Model, *Anal. Chem.*, 2016, **88**, 5610–5614.

25 Q. Yang, S. Wang, D. Li, J. Yuan, J. Xu and S. Shao, A mitochondria-targeting nitroreductase fluorescent probe with large Stokes shift and long-wavelength emission for imaging hypoxic status in tumor cells, *Anal. Chim. Acta*, 2020, **1103**, 202–211.

26 W. Wanas, S. A. Abd El-Kaream, S. Ebrahim, M. Soliman and M. Karim, Cancer bioimaging using dual mode luminescence of graphene/FA-ZnO nanocomposite based on novel green technique, *Sci. Rep.*, 2023, **13**, 27.

27 B. Brennecke, Q. Wang, Q. Zhang, H. Y. Hu and M. Nazaré, An Activatable Lanthanide Luminescent Probe for Time-Gated Detection of Nitroreductase in Live Bacteria, *Angew. Chem. Int. Ed.*, 2020, **59**, 8512–8516.

28 Q. Yang, Y. Wen, A. Zhong, J. Xu and S. Shao, An HBT-based fluorescent probe for nitroreductase determination and its application in: Escherichia coli cell imaging, *New J. Chem.*, 2020, **44**, 16265–16268.

29 H. Jiang, Z. Cao, Y. Liu, R. Liu, Y. Zhou and J. Liu, Bacteria-Based Living Probes: Preparation and the Applications in Bioimaging and Diagnosis, *Adv. Sci.*, 2024, **11**, 2306480.

30 Y. Fan, M. Lu, X. A. Yu, M. He, Y. Zhang, X. N. Ma, J. Kou, B. Y. Yu and J. Tian, Targeted Myocardial Hypoxia Imaging Using a Nitroreductase-Activatable Near-Infrared Fluorescent Nanoprobe, *Anal. Chem.*, 2019, **91**, 6585–6592.

31 J. Zheng, Y. Shen, Z. Xu, Z. Yuan, Y. He, C. Wei, M. Er, J. Yin and H. Chen, Near-infrared off-on fluorescence probe activated by

 This journal is © The Royal Society of Chemistry 20xx

Please do not adjust margins

Please do not adjust margins

NTR for in vivo hypoxia imaging, *Biosens. Bioelectron.*, 2018, **119**, 141–148.

32 X. Han, R. Wang, X. Song, F. Yu, C. Lv and L. Chen, A mitochondrial-targeting near-infrared fluorescent probe for bioimaging and evaluating endogenous superoxide anion changes during ischemia/reperfusion injury, *Biomaterials*, 2018, **156**, 134–146.

33 J. Qian and B. Z. Tang, AIE Luminogens for Bioimaging and Theranostics: From Organelles to Animals, *Chem*, 2017, **3**, 56–91.

34 C. Pigot, D. Brunel and F. Dumur, Indane-1,3-Dione: From Synthetic Strategies to Applications, *Molecules*, 2022, **27**, 5976.

35 S. M. Bentzen, Theragnostic imaging for radiation oncology: dose-painting by numbers, *Lancet Oncol*, 2005, **6**, 112–117.

36 M. J. Mantilla, C. E. Cabrera Díaz, G. Ariza-Aranguren, H. de Cock, J. B. Helms, S. Restrepo, E. Jiménez and A. M. Celis Ramírez, Back to the Basics: Two Approaches for the Identification and Extraction of Lipid Droplets from *Malassezia pachydermatis* CBS1879 and *Malassezia globosa* CBS7966, *Curr. Protoc.*, 2021, **1**, e122.

37 D. Liang, C. Yu, X. Qin, X. Yang, X. Dong, M. Hu, L. Du and M. Li, Discovery of small-molecule fluorescent probes for C-Met, *Eur. J. Med. Chem.*, 2022, **230**, 114114.

38 A. Tigreros, M. Macías and J. Portilla, Structural, Photophysical, and Water Sensing Properties of Pyrazolo[1,5-a]pyrimidine-Triphenylamine Hybrid Systems, *ChemPhotoChem*, 2022, **2022**, e202200133.

39 D. Cao, Z. Liu, P. Verwilst, S. Koo, P. Jangjili, J. S. Kim and W. Lin, Coumarin-Based Small-Molecule Fluorescent Chemosensors, *Chem. Rev.*, 2019, **119**, 10403–10519.

40 J. Xie, L. Wang, X. Su and J. Rodrigues, Coumarin-based Fluorescent Probes for Bioimaging: Recent Applications and Developments, *Curr. Org. Chem.*, 2021, **25**, 2142–2154.

41 R. J. Grams, W. L. Santos, I. R. Scorei, A. Abad-García, C. A. Rosenblum, A. Bita, H. Cerecetto, C. Viñas and M. A. Soriano-Ursúa, The Rise of Boron-Containing Compounds: Advancements in Synthesis, Medicinal Chemistry, and Emerging Pharmacology, *Chem. Rev.*, 2024, **124**, 2441–2511.

42 S. Das, S. Dey, S. Patra, A. Bera, T. Ghosh, B. Prasad, K. D. Sayala, K. Maji, A. Bedi and S. Debnath, BODIPY-Based Molecules for Biomedical Applications, *Biomolecules*, 2023, **13**, 1723.

43 M. C. Rios, N. F. Bravo, C. C. Sanchez and J. Portilla, Chemosensors based on N-heterocyclic dyes: Advances in sensing highly toxic ions such as CN- and Hg2+, *RSC Adv.*, 2021, **11**, 34206–34234.

44 J. T. Sarmiento and J. Portilla, Current Advances in Diazoles-based Chemosensors for CN- and FDetection, *Curr. Org. Synth.*, 2023, **20**, 77–95.

45 J. Zhang, X. Chai, X.-P. He, H.-J. Kim, J. Yoon and H. Tian, Fluorogenic probes for disease-relevant enzymes, *Chem. Soc. Rev.*, 2019, **48**, 683–722.

46 Y. Yang, Q. Zhao, W. Feng and F. Li, Luminescent Chemodosimeters for Bioimaging, *Chem. Rev.*, 2013, **113**, 192–270.

47 C. L. Fleming, P. A. Sandoz, T. Inghardt, B. Önfelt, M. Grøtli and J. Andréasson, A Fluorescent Kinase Inhibitor that Exhibits Diagnostic Changes in Emission upon Binding, *Angew. Chem. Int. Ed.*, 2019, **58**, 15000–15004.

48 L. Türker and S. Variş, A review of polycyclic aromatic energetic materials, *Polycycl. Aromat. Compd.*, 2009, **29**, 228–266.

49 M. Stępień, E. Gońka, M. Żyła and N. Sprutta, Heterocyclic Nanographenes and Other Polycyclic Heteroaromatic Compounds: Synthetic Routes, Properties, and Applications, *Chem. Rev.*, 2017, **117**, 3479–3716.

50 H. M. Kim and B. R. Cho, Small-Molecule Two-Photon Probes for Bioimaging Applications, *Chem. Rev.*, 2015, **115**, 5014–5055.

51 A. Loudet and K. Burgess, BODIPY Dyes and Their Derivatives: Syntheses and Spectroscopic Properties, *Chem. Rev.*, 2007, **107**, 4891–4932.

52 M. O. Senge, N. N. Sergeeva and K. J. Hale, Classic highlights in porphyrin and porphyrinoid total synthesis and biosynthesis, *Chem. Soc. Rev.*, 2021, **50**, 4730–4789.

53 J. Hwang, T. S. Reddy, H. Moon, H. D. Lee and M.-S. Choi, Cyanide detecting porphyrin fluorescent sensors: Effects of electron-donating/withdrawing substituents, *Dyes Pigm.*, 2023, **215**, 111243.

54 A. L. Pang, A. Arsad and M. Ahmadipour, Synthesis and factor affecting on the conductivity of polypyrrole: a short review, *Polym. Adv. Technol.*, 2021, **32**, 1428–1454.

55 F.-Z. Li, J.-F. Yin and G.-C. Kuang, BODIPY-based supramolecules: Construction, properties and functions, *Coord. Chem. Rev.*, 2021, **448**, 214157.

56 D. Kim, D. Ma, M. Kim, Y. Jung, N. H. Kim, C. Lee, S. W. Cho, S. Park, Y. Huh, J. Jung and K. H. Ahn, Fluorescent Labeling of Protein Using Blue-Emitting 8-Amino-BODIPY Derivatives, *J. Fluoresc.*, 2017, **27**, 2231–2238.

57 M. L. S. O. Lima, C. B. Braga, T. B. Becher, M. Odriozola-Gimeno, M. Torrent-Sucarrat, I. Rivilla, F. P. Cossío, A. J. Marsaioli and C. Ornelas, Fluorescent Imidazo[1,2-a]pyrimidine Compounds as Biocompatible Organic Photosensitizers that Generate Singlet Oxygen: A Potential Tool for Phototheranostics, *Chem. Eur. J.*, 2021, **27**, 6213–6222.

58 A. Kolbus, T. Uchacz, A. Danel, K. Gałczyńska, P. Moskwa and P. Kolek, Fluorescent Sensor Based on 1H-Pyrazolo[3,4-b]quinoline Derivative for Detecting Zn2+ Cations, *Molecules*, 2024, **29**, 823.

Please do not adjust margins

Please do not adjust margins

59 J. Orrego-Hernández, C. Lizarazo, J. Cobo and J. Portilla, Pyrazolo-fused 4-azafluorenones as key reagents for the synthesis of fluorescent dicyanovinylidene-substituted derivatives, *RSC Adv.*, 2019, **9**, 27318–27323.

60 K. Saravana Mani, R. Rajamanikandan, G. Ravikumar, B. Vijaya Pandiyan, P. Kolandaivel, M. Ilanchelian and S. P. Rajendran, Highly Sensitive Coumarin–Pyrazolone Probe for the Detection of Cr^{3+} and the Application in Living Cells, *ACS Omega*, 2018, **3**, 17212–17219.

61 S. B. Subramaniyan, S. B. Annes, M. Yuvasri, K. Nivedha, S. Ramesh and V. Anbazhagan, 1,3,5-Triphenylpyrazoline Based Fluorescent Probe for Selective Sensing and Imaging of Glutathione in Live Cell under Oxidative Stress, *ChemistrySelect*, 2020, **5**, 515–521.

62 A. Tigreros and J. Portilla, Recent progress in chemosensors based on pyrazole derivatives, *RSC Adv.*, 2020, **10**, 19693–19712.

63 M.-C. Ríos and J. Portilla, Recent Advances in Synthesis and Properties of Pyrazoles, *Chemistry (Easton)*, 2022, **4**, 940–968.

64 J. Portilla, Current Advances in Synthesis of Pyrazole Derivatives: An Approach Toward Energetic Materials, *J. Heterocycl. Chem.*, , DOI:10.1002/jhet.4904.

65 J. Orrego-Hernández, J. Cobo and J. Portilla, Synthesis, Photophysical Properties, and Metal-Ion Recognition Studies of Fluoroionophores Based on 1-(2-Pyridyl)-4-Styrylpyrazoles, *ACS Omega*, 2019, **4**, 16689–16700.

66 M. U and S. S, A review on pyrazole moieties as organic chemosensors in the detection of cations and anions, *Inorg. Chim. Acta*, 2024, **569**, 122118.

67 X. B. Wang, H. J. Li, Z. Chi, X. Zeng, L. J. Wang, Y. F. Cheng and Y. C. Wu, A novel mitochondrial targeting fluorescent probe for ratiometric imaging SO2 derivatives in living cells, *J. Photochem. Photobiol. A*, 2020, **390**, 112339.

68 E. Janczy-Cempa, O. Mazuryk, D. Sirbu, N. Chopin, M. Żarnik, M. Zastawna, C. Colas, M. A. Hiebel, F. Suzenet and M. Brindell, Nitro-Pyrazinotriazapentalene scaffolds– nitroreductase quantification and in vitro fluorescence imaging of hypoxia, *Sens. Actuators B: Chem.*, 2021, **346**, 130504.

69 S. Roy, S. Kundu, S. Saha, K. Muddukrishnaiah, R. Pramanik and B. Biswas, Visible light-triggered pyrazole-functionalized reversible ionophore for selective monitoring of aluminium (III) ion, *Appl. Organomet. Chem.*, 2022, **36**, e6865.

70 G. J. Shi, Y. D. Wang, Z. X. Yu, Q. Zhang, S. Chen, L. Z. Xu, K. P. Wang and Z. Q. Hu, The coumarin-pyrazole dye for detection of hydrogen sulfide in cells, *Spectrochim. Acta - A: Mol. Biomol.*, 2023, **285**, 121898.

71 P. Rasin, S. M. Basheer, J. Haribabu, K. N. Aneesrahman, V. Manakkadan, V. N. Vadakkedathu Palakkeezhillam, N. Bhuvanesh, C. Echeverria, J. F. Santibanez and A. Sreekanth, Host-guest interactions of coumarin-based 1,2-pyrazole using analytical and computational methods: Paper strip-based detection, live cell imaging, logic gates and keypad lock applications, *Heliyon*, 2024, **10**, e24077.

72 Y. Zhang, X. Zheng, L. Zhang, Z. Yang, L. Chen, L. Wang, S. Liu and Z. Xie, Red fluorescent pyrazoline-BODIPY nanoparticles for ultrafast and long-term bioimaging, *Org. Biomol. Chem.*, 2020, **18**, 707–714.

73 L. R. V. Favarin, G. B. Laranjeira, C. F. A. Teixeira, H. Silva, A. C. Micheletti, L. Pizzuti, A. Machulek Júnior, A. R. L. Caires, V. M. Deflon, R. B. P. Pesci, C. N. L. Rocha, J. R. Correa, L. M. C. Pinto and G. A. Casagrande, Harvesting greenish blue luminescence in gold(i) complexes and their application as promising bioactive molecules and cellular bioimaging agents, *New J. Chem.*, 2020, **44**, 6862–6871.

74 A. Alizadeh, B. Farajpour, S. S. Mohammadi, M. Sedghi, H. Naderi-Manesh, C. Janiak and T. Knedel, Design and Synthesis of Coumarin-Based Pyrazolopyridines as Biocompatible Fluorescence Dyes for Live-Cell Imaging, *ChemistrySelect*, 2020, **5**, 9362–9369.

75 X. Wang, L. Wang, L. Xie, Z. Xie, L. Li, D. Bui, T. Yin, S. Gao and M. Hu, Design and Synthesis of a Novel NIR Celecoxib-Based Fluorescent Probe for Cyclooxygenase-2 Targeted Bioimaging in Tumor Cells, *Molecules*, 2020, **25**, 4037.

76 X. He, X. Cao, X. Tian and Y. Bai, A simple fluorescent probe for glutathione detection and its bioimaging application in living cells, *Microchem. J.*, 2021, **166**, 106135.

77 Y. Huang, Y. Zhang, F. Huo and C. Yin, An innovative hypochlorite-sensing scaffold and its imaging application in vivo, *Dyes Pigm.*, 2021, **191**, 109378.

78 K. Krishnaveni, S. Murugesan and A. Siva, Fluorimetric and colorimetric detection of multianalytes Zn2+/Cd2+/F− ions via 5-bromosalicyl hydrazone appended pyrazole receptor; live cell imaging analysis in HeLa cells and zebra fish embryos, *Inorg. Chem. Commun.*, 2021, **132**, 108843.

79 H. Hosseinjani-Pirdehi, N. O. A. Mahmoodi and A. Taheri, Selective Cu2+ detection by a novel fluorescence hydrazone – Base probe in aqueous media, *J. Photochem. Photobiol. A*, 2021, **421**, 113524.

80 R. Bag, Y. Sikdar, S. Sahu, C. Das Mukhopadhyay, M. G. B. Drew and S. Goswami, Benzimidazole based ESIPT active chemosensors enable nano–molar detection of Cu2+ in 90% aqueous solution, MCF–7 cells, and plants, *J. Photochem. Photobiol. A*, 2022, **431**, 114006.

81 K. M. Kuznetsov, V. A. Baigildin, A. I. Solomatina, E. E. Galenko, A. F. Khlebnikov, V. V. Sokolov, S. P. Tunik and J. R. Shakirova, Polymeric Nanoparticles with Embedded Eu(III) Complexes as Molecular Probes for Temperature Sensing, *Molecules*, 2022, **27**, 8813.

82 U. Krishnan, S. Manickam and S. K. Iyer, BF3 detection by pyrazolo-pyridine based fluorescent probe and applications in bioimaging and paper strip analysis, *J. Mol. Liq.*, 2023, **385**, 122413.

This journal is © The Royal Society of Chemistry 20xx

Please do not adjust margins

Please do not adjust margins

83 Y. S. Yang, F. N. Wang, Y. P. Zhang, F. Yang and J. J. Xue, Novel Bis-pyrazoline Fluorescent Probe for Cu2+ and Fe3+ Detection and Application in Cell Imaging, *J. Fluoresc.*, 2024, **34**, 159–167.

84 K. Wei, B. Zhang, Y. Liu, M. Kang, P. Liu, X. Yang, M. Pei and G. Zhang, Comparison of two pyrazole derived “turn on” fluorescent probes for the recognition of Ga3+, *J. Photochem. Photobiol. A*, 2023, **440**, 114656.

85 N. Patil, R. Dhake, R. Phalak, U. Fegade, C. Ramalingan, V. Saravanan, Inamuddin and T. Altalhi, A Colorimetric Distinct Color Change Cu(II) 4-{[1-(2,5-dihydroxyphenyl)ethylidene]amino}-1,5-dimethyl-2-phenyl-1,2-dihydro-3H-pyrazol-3-one Chemosensor and its Application as a Paper Test Kit, *J. Fluoresc.*, 2023, **33**, 1089–1099.

86 X. Li, Z. Meng, S. Gong, Y. Liang, Y. Zhang, Y. Yang, X. Xu, Z. Wang and S. Wang, Development of a novel ratiometric fluorescent probe for real-time monitoring of acidic pH and its applications in food samples and biosystem, *Microchem. J.*, 2024, **204**, 111169.

87 X. Liu, K. Wang, L. Wei, Y. Wang, C. Liu, X. Rong, T. Yan, W. Shu and B. Zhu, A highly sensitive Golgi-targeted fluorescent probe for the simultaneous detection of malondialdehyde and formaldehyde in living systems and foods, *Talanta*, 2024, **278**, 126427.

88 Y. Deng, T. Shen, X. Yu, J. Li, P. Zou, Q. Gong, Y. Zheng, H. Sun, X. Liu and H. Wu, Tetrazine-Isonitrile Bioorthogonal Fluorogenic Reactions Enable Multiplex Labeling and Wash-Free Bioimaging of Live Cells, *Angew. Chem. Int. Ed.*, 2024, **63**, e2023198.

89 C. Krishnamurthy, K. Jathi, K. M. Pallavi and C. Yesudhasan, Hydrazo Pyrazole-Pyridone Fluorescent tag for NLO, Live cell imaging, LFPs visualization, Photophysical probing, and Electrochemical sensor for Dopamine detection, *Luminescence*, 2024, **39**, e4760.

Please do not adjust margins
